# Genes for Membrane Transport Proteins: Not So Rare in Viruses

**DOI:** 10.3390/v10090456

**Published:** 2018-08-26

**Authors:** Timo Greiner, Anna Moroni, James L Van Etten, Gerhard Thiel

**Affiliations:** 1 University Clinic for Psychiatry and Psychotherapy, Brandenburg Medical School, Immanuel Klinik Rüdersdorf, 15562 Rüdersdorf, Germany; 2 Department of Biosciences, University of Milan, 20133 Milan, Italy; anna.moroni@unimi.it; 3 Department of Plant Pathology and Nebraska Center for Virology, University of Nebraska, Lincoln, NE 68583-0900, USA; jvanetten1@unl.edu; 4 Membrane Biophysics, Department of Biology, Technische Universität Darmstadt, 64287 Darmstadt, Germany; thiel@bio.tu-darmstadt.de

**Keywords:** Phycodnaviridae, algae viruses, Mimiviridae, ion channels, transporter, ATPase, virus evolution

## Abstract

Some viruses have genes encoding proteins with membrane transport functions. It is unknown if these types of proteins are rare or are common in viruses. In particular, the evolutionary origin of some of the viral genes is obscure, where other viral proteins have homologs in prokaryotic and eukaryotic organisms. We searched virus genomes in databases looking for transmembrane proteins with possible transport function. This effort led to the detection of 18 different types of putative membrane transport proteins indicating that they are not a rarity in viral genomes. The most abundant proteins are K^+^ channels. Their predicted structures vary between different viruses. With a few exceptions, the viral proteins differed significantly from homologs in their current hosts. In some cases the data provide evidence for a recent gene transfer between host and virus, but in other cases the evidence indicates a more complex evolutionary history.

## 1. Introduction

Following the discovery that the M2 protein of influenza virus A functions as a H^+^ channel [1], other viruses were discovered that also code for membrane transport proteins [2,3,4,5]. These proteins can be divided into two groups: (i) channels/transporters unique to viruses, i.e., without homologs in eukaryotes or prokaryotes and (ii) proteins with homologs in eukaryotic and prokaryotic organisms. The former group of unique viral channels and transporters has been reviewed by others [5,6]. This group is known as viroporins or—more generally—as viral ion channels. Well-studied examples in this group are the aforementioned M2 protein from influenza A [1,7,8] and Vpu from HIV-1 [9,10,11]. The group of viral proteins with homologs in eukaryotes/prokaryotes is best characterized by the K^+^ channel Kcv from chloroviruses, a protein, which has the major structural and functional features of K^+^ channels from prokaryotic and eukaryotic organisms [12,13].

An alternative way to classify viral transport proteins is by their necessity for virus replication and their abundance within a virus family/order. Both, M2 and Kcv, for example, are found in all influenza and most chlorella virus species, respectively [1,14]. Both proteins are packaged in the virus particles and they aid infection of the host or serve distinct roles in viral replication [15,16,17]. The functional role of other viral transport proteins is more uncertain. In the case of the chloroviruses, some of these viruses also encode transmembrane (TM) proteins, such as potassium transporters, calcium transporting adenosine triphosphatases (ATPases), and aquaglyceroporins [14]. Even though it was shown that these proteins are functional ([18,19,20]; Table S1) their role(s) in virus infection/replication is unknown. The viruses, which encode these transport proteins in addition to the K^+^ channel, show no obvious growth advantages in the laboratory over those without these proteins.

The presence of virus-encoded transport proteins raises several interesting questions. One obvious question concerns the evolutionary relationship between homologs of transport proteins in viruses and eukaryotes and prokaryotes. This question is of particular interest considering the ongoing debate about the origin of large DNA viruses and their genes [21,22,23]. In a previous analysis it was reported that the K^+^ channel proteins encoded by different algal viruses were more similar to each other than to any of the K^+^ channel proteins encoded by their hosts [24]. This finding, plus other data, led to the suggestion that the viral K^+^ channels might be the evolutionary ancestor of prokaryotic and eukaryotic K^+^ channels [25]. While this conclusion is supported by a small dataset from a few viral channels and the channels of two hosts, it is not clear if this concept holds for a larger set of data. A second question concerns the potential role of diverse membrane transport proteins in the viral infection/replication cycle. With the assumption that viruses do not normally carry “useless” genes, we anticipate that all viral-encoded transport proteins eventually play some role during infection/replication, and that their activity may be somewhat modified, or even optimized, for this function. An interesting example of this phenomenon has been reported for a different type of protein: the phage of a *Prochlorococcus* species carries two genes for photosynthesis. The host, which utilizes three genes for the same process shuts down its photosynthetic processes when infected. The phage rescues this activity by using its own gene products with higher efficiency [26]. The phage has thus optimized the process so much that it even omits one of the proteins, which is required in the cellular machinery. Functional optimization is not necessarily the only goal a virus might pursue when modifying proteins.

With more viral genomes becoming available we performed database searches for different types of viral encoded membrane transport proteins. This data mining provided evidence for a wide range of different channel and transporter proteins in many viral genomes. We use this information to compare the viral proteins to homologs from eukaryotic and prokaryotic organisms, including the hosts of the viruses. A search for similarities and differences between the viral genes and their putative current or former hosts provided information on the possible origins of the viral genes under the assumption that they were acquired from the virus’ host.

## 2. Materials and Methods

We searched for viral membrane transport proteins using three different strategies: (i) We screened the NCBI protein database [27] for viral genes that were annotated as “channel”, “transporter”, “exchanger”, “symporter”, “antiporter”, “ATPase”, or “pump”. (ii) We searched virus genome annotations for the same keywords as in i. (iii) We used the blastp search [28] against viral sequences to find homologs of known channel and transporter proteins from eukaryotes and prokaryotes. We then evaluated the proteins for possible channel or transporter function using the following criteria: (i) presence of conserved domains, which are crucial for the function of the proteins and (ii) the presence of at least one TM domain. TM domains were predicted with the TMHMM algorithm [29].

To obtain clues about the possible origins of the viral proteins, we performed the following: (i) we again used the blastp search against the whole database in order to identify the most similar proteins and (ii) we used MEGA software version 7 [30] to construct phylogenetic trees with viral, eukaryotic, and prokaryotic protein sequences of the same type.

Lastly, we made sequence alignments in order to compare viral and homologous sequences to identify the main differences between membrane transport proteins from viruses and from cellular organisms.

The yeast complementation assay to study potassium fluxes has been described elsewhere [31]. PCR using Phusion DNA polymerase (New England Biolabs GmbH, Frankfurt/Main, Germany) was performed to amplify viral genes using primers containing specific restriction sites. A diluted virus suspension was directly added to the PCR reaction mixture. The reaction was then loaded onto an agarose gel and the amplified DNA fragments were cut from the gel and purified for cloning. Viral genes, which encode putative transport proteins were transformed into SGY1528 yeast strain (Mat a ade2–1 can1–100 his3–11,15 leu2–3,112 trp1–1 ura3–1 trk1::HIS3 trk2::TRP1), which is deficient in endogenous K^+^ uptake systems. Yeasts from the same stock were grown in parallel under nonselective conditions on plates containing 100 mM KCl and on selective conditions on agar containing 0.5 mM KCl. Growth experiments were conducted at 30 °C.

Electrophysiological recordings from Human embryonic kidney 293 (HEK293) cells (from American Type Culture Collection) were obtained using the patch-clamp technique in cell-attached configuration. Patch-clamp pipettes were pulled from thick-walled borosilicate capillaries (with filament; Harvard Apparatus, Edenbridge, UK) and tips were fire polished to obtain a final pipette resistance of 8–12 MΩ. In addition, pipettes were coated near the tip with Sylgard (Dow Corning, Coventry, UK).

The bath solution contained (in mM): 145 NaCl, 5 KCl, 2 CaCl_2_, 1.2 MgCl_2_, 5 HEPES, and 30 glucose; the pH was adjusted to 7.4 with NaOH. The pipette solution was identical to the extracellular solution but with agonist added to the required concentration. All solutions were prepared from double-distilled water and filtered through a 0.2 µm Cyclopore track-etched membrane (GE Healthcare life sciences, Buckinghamshire, UK) to remove impurities.

For expression, cells were plated on poly-l-lysine coated glass coverslips (Sigma-Aldrich, Gillingham, UK and VWR, Leicestershire, UK, respectively) in 35 mm culture dishes (Scientific Laboratory Supplies, SLS, Nottingham, UK) containing 2 mL Dulbecco’s Modified Eagle’s Medium (DMEM), Gibco, (Thermo Fisher Scientific, Paisley, UK), and were then transfected via the calcium phosphate-precipitation method with pcDNA3.1 plasmids coding for the genes of interest. A plasmid coding for the enhanced Green Fluorescent Protein (eGFP) was added to allow detection of transfected cells. The final DNA mixture contained 2% virus channel cDNA, 20% eGFP cDNA and 78% empty pcDNA3.1 plasmid. The empty plasmid was added to avoid overexpression of the viral gene. The total amount of final DNA mixture was 3 μg per plate. The transfection medium was washed off and replaced by fresh DMEM 4 h after transfection. Electrophysiological experiments were performed one day after transfection.

Currents were recorded with an Axopatch 200B amplifier (MDS Analytical Technologies, Molecular Devices, Sunnydale, CA, USA). Recordings were pre-filtered at 10 KHz with a four-pole low-pass Bessel filter (built in the amplifier), digitized with a Digidata 1322A (MDS Analytical Technologies; sampling rate 100 kHz) and stored directly on the computer hard drive via Clampex 10.2 software (MDS Analytical Technologies).

## 3. Results

Overall, our search criteria, which are detailed in the Materials and Methods, resulted in the discovery of 18 different types of putative virus-encoded membrane transport proteins. These are: (i) K^+^ channels from phycodnaviruses, a mimivirus and various phages; (ii) two types of chloride channels from herpesviruses; (iii) a glutamate receptor from chloroviruses; (iv) ligand-gated channels from chloroviruses; (v) an aquaglyceroporin from chloroviruses; (vi) three different mechanosensitive channels from large DNA viruses; (vii) an ammonium transporter from a prasinovirus; (viii) Mg^2+^ transporters from phages; (ix) sodium phosphate transporters from coccolithoviruses; (x) sodium glucose transporters from phages; (xi) sodium calcium symporters from phages; (xii) two types of ATP-binding casette (ABC) transporters from mimiviruses and from entomopoxviruses; (xiii) nucleotide transporters from phages; (xiv) nucleoside transporters from herpesviruses; (xv) amino acid transporters from iridoviruses; (xvi) a Ca^2+^ transporting ATPase from chloroviruses; (xvii) a Mg^2+^ transporting ATPase from a mimivirus; and (xviii) a p-type ATPase from a mimivirus. Details are summarized in Table S2.

The respective genes belonged mostly to large DNA viruses including members of the Herpesviridae, Mimiviridae, Poxviridae and Phycodnaviridae, as well as phages in the order Caudovirales and therein the families Siphoviridae and Myoviridae. The smallest genome among these viruses, which carry membrane transport genes, belonged to Lactobacillus phage PLE (35,068 bp) and the largest genome belonged to Tupanvirus from the deep ocean (1,516,267 bp).

### 3.1. Channels

#### 3.1.1. Potassium Channels

**Figure 1:**
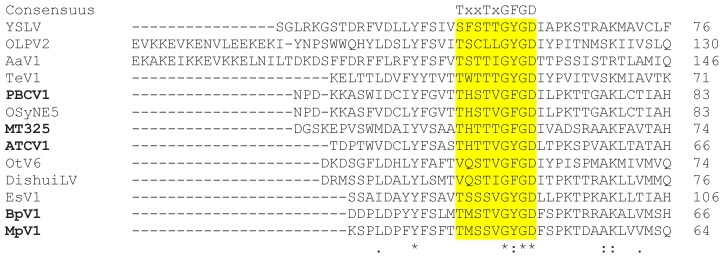
Alignment of K^+^ channel consensus domain in alga virus K^+^ channel proteins. Alignment of known alga virus K^+^ channels from the following viruses: Paramecium bursaria chlorella virus-1 (PBCV-1), Only Syngen chlorella virus (OSy-NE5), Ectocarpus siliculosus virus-1 (EsV-1), Chlorella Pbi virus MT325 (MT325), Acanthocystis turfacea chlorella virus-1 (ATCV-1), Bathycoccus sp. RCC1105 virus (BpV1), Micromonas sp. RCC1109 virus (MpV1), Ostreococcus tauri virus RT-2011 (OtV6), Dishui Lake phycodnavirus 1 (DishuiLV), Yellowstone Lake phycodnavirus (YSLV), Aureococcus anophagefferens virus-1 (AaV1), Tetraselmis virus-1 (TeV-1), and Organic Lake Phycodnavirus-2 (OLPV2). Channels with experimentally confirmed function are shown in bold. The conserved filter region is highlighted in yellow; the complete alignment is shown in Figure S1A. In the alignment, identical amino acids are indicated by “*”, conserved and semi-conserved amino acids are indicated by “:” and “.”, respectively.

**Figure 2:**
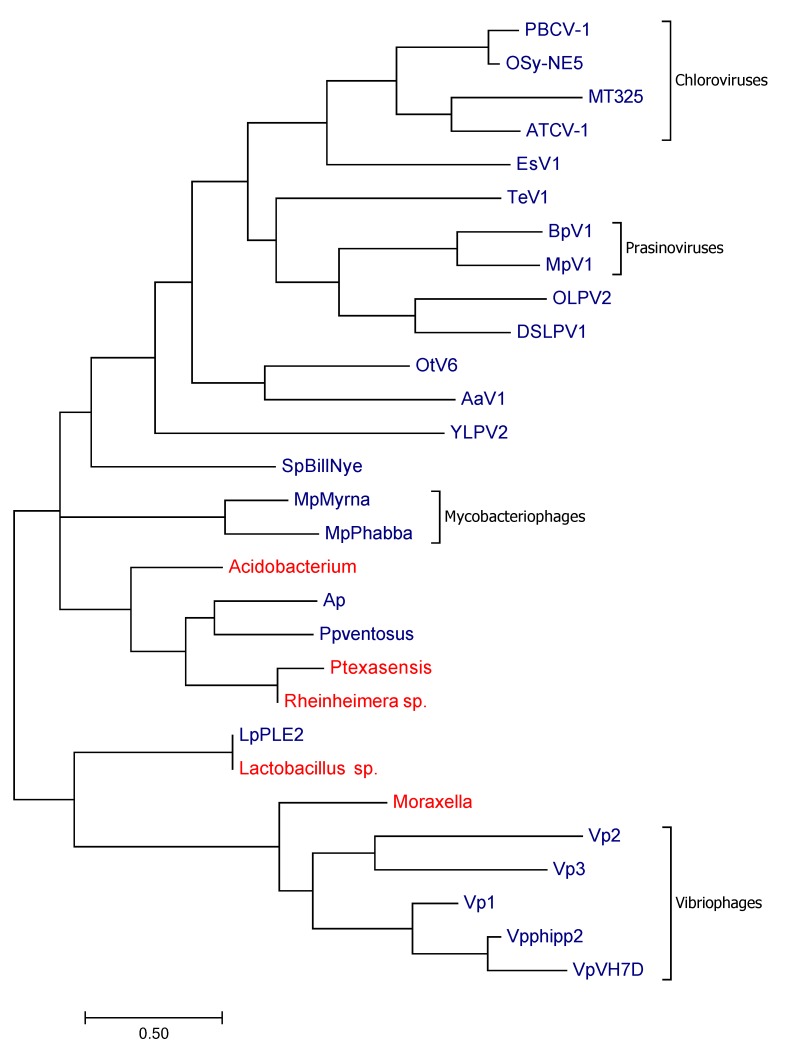
Maximum likelihood tree of K^+^ channel proteins. The tree includes sequences from viruses (blue) and sequences from cellular organisms (red) that were most similar to the viral sequences. A possible evolutionary history of the diverse channels is inferred from the maximum likelihood method based on the JTT matrix-based model [38]. Here we show the tree with the highest log likelihood (−3715.28). The tree was obtained as described in [38] from a combination of neighbor-joining and BioNJ algorithms. The tree is presented in such a way that the branch lengths are a measure for the number of substitutions per site. The analysis is based on 29 protein sequences. All positions containing gaps and missing data were eliminated. In the present case we considered a total of 69 positions in the final dataset. For the evolutionary analyses we used MEGA7 software. The alignment for the sequences used in this tree is shown in Figure S1B.

Potassium channels are proteins that facilitate the transport of K^+^ across membranes. They contain at least two TMs and a conserved sequence motif that acts as a filter for selecting K^+^ over Na^+^ [32]. Potassium channels are the most abundant viral membrane transport proteins and they are detected in very different viruses and phages. All of them contain the highly conserved signature sequence G(Y/F)GD and at least two predicted TM domains. In addition to the already known K^+^ channels from the chloroviruses, prasinoviruses, the phaeovirus Ectocarpus siliculosus virus-1 (EsV-1), and two other phycodnaviruses (see Table S1), four more phycodnavirus genes resembling K^+^ channels were found. One channel gene was detected in the recently described chlorovirus OSy-NE5 [33] (94 amino acids (aa), 2 predicted TMs, accession #YP_009325597.1) and two genes were found in viruses from environmental samples, Dishui Lake phycodnavirus, and Yellowstone Lake phycodnavirus (116 aa, two predicted TMs, accession #AUT19143.1, and 96 aa, two predicted TMs, NCBI accession #YP_009174599.1). An additional gene was found in the newly discovered Tetraselmis virus-1 [34] (TeV-1; 81 aa, two predicted TMs, accession #AUF82121.1). The alignment of the K^+^ channel consensus domain (Figure 1, full alignment in Figure S1) and the corresponding phylogenetic tree in Figure 2 show that the similarity to homologous K^+^ channel sequences was low for all four additional proteins. All channels contain the typical G(Y/F)G motive in the selectivity filter and a subsequent Asp, which is common in this position [35]. Outside of this domain the channels exhibit some diversity from the canonical K^+^ channel consensus sequence although a Thr/Ser [35] or a Thr/Val substitution has previously been reported for some other two pore domain K^+^ channels. Variability from canonical K^+^ channels is also apparent with respect to the pair of aromatic amino acids, which are generally present upstream of the selectivity filter in K^+^ channels [36]. They are not present in all sequences, however, and their absence does not prevent channel function in the case of the protein from virus MT325 [18].

The blastp E values for the most similar hits were 4e−6 (OSy-NE5), 8e−8 (Dishui Lake phycodnavirus), 2e−7 (Yellowstone Lake phycodnavirus) and 2e−7 (TeV-1). The diversity of all phydcodnavirus K^+^ channels is high as indicated by the alignment in Figure S1 and the corresponding phylogenetic tree (Figure 2). The proteins not only vary in size ranging from 79 to 156 amino acids in length, but also in overall amino acid composition.

Potassium ion channel encoding genes were also discovered in phage genomes. At least 1600 genomes of mycobacteriophages have been completely sequenced [37] and in two of them we detected a K^+^ channel encoding gene: one in Mycobacterium phage Myrna (119 aa, two predicted TMs, accession #ACH62227.1) and one in Mycobacterium phage Phabba (119 aa, two predicted TMs, accession #ASZ74807.1). The two genes are very similar to each other (70% aa identity). The most similar non-viral protein is a protein, which is annotated as an “ion transport protein” from *Acidobacteria bacterium* OLB7 (blastp E value 4e−19, 38% aa identity, accession #KXK00635.1).

**Figure 3:**
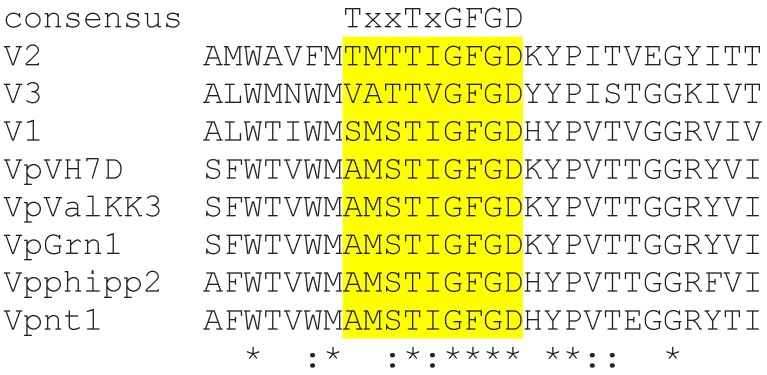
Putative K^+^ channel proteins from vibrio phages. Alignment of the K^+^ channel consensus domain from vibrio phage K^+^ channel sequences. The conserved signature motif (T/S)T(I/V)GFGD is highlighted in yellow. Viruses: Vibrio phage phi-pp2 (Vpphipp2), Vibrio phage nt-1 (Vpnt1), Vibrio phage ValKK3 (VpValKK3), Vibrio phage VH7D (VpVH7D), Vibrio phage phi-Grn1 (VpGrn1), Vibrio phage phi-ST2 (VpST2), Vibrio phage 1.081.O._10N.286.52.C2 (Vp1), Vibrio phage 1.084.O._10N.261.49.F5 (Vp2), and Vibrio phage 2.275.O._10N.286.54.E11 (Vp3). The complete alignment is shown in Figure S2A. In the alignment, identical amino acids are indicated by “*”, conserved and semi-conserved amino acids are indicated by “:” and “.”, respectively.

At least nine Vibrio phages carry genes coding for a putative K^+^ channel protein with the consensus sequence of K^+^ channels (Figure 3). The similarity in this domain and the agreement with the canonical K^+^ channel consensus sequence is quite high in this domain. Additionally, the typical aromatic amino acids upstream of the filter sequence [36] are present. Diversity is only apparent in the first position of the consensus sequence, which is frequently, but not necessarily, a Thr in K^+^ channels. The phage proteins vary in size from 149 to 228 amino acids, but exhibit the overall architecture of K^+^ channel proteins (Table S3 and Figure S2A). Interesting to note is that the putative channel protein of Vibrio phage phi-ST2 lacks the first TM domain. This does not exclude not per se a K^+^ channel function for these proteins. The conventional algorithms are optimized for predictions of structural domains in eukaryotes and prokaryotes and may fail to detect existing TM domains in viral proteins [39]. We also found in the case of the chlorovirus AR158 Kcv that the protein was much shorter than its homologs and that the outer TM domain was lacking. We do not know how this finding fits into the general picture in which the viral channels seem to be essential for replication. At this point we speculate that the function of the truncated channel proteins might be restored by a separately, but unknown, virus or host encoded protein.

A blastp search of the Vibrio phage phi-pp2 protein against non-viral organisms revealed a putative K^+^ channel protein from a Moraxellaceae bacterium as the most similar protein (E value 2e−26, 44% aa identity; accession #PCJ41331.1). The similarity between the viral and bacterial protein however is rather low and, interestingly, the viral protein is longer than the bacterial one (228 vs. 158 aa; Figure S2B).

Additional K^+^ channel genes were found in *Acinetobacter phage* vB_AbaM_ME3 (124 aa, 2 TMs, accession #AND75308.1) and Pseudomonas phage ventosus (117 aa, 2 TMs, accession #ATW58311.1). Both proteins slightly resemble a hypothetical protein from *Rheinheimera* sp. F8 (accession #ALZ75942.1). The blastp E values and aa identities were 2e−28 and 41% (Acinetobacter phage) and 1e−33 and 44% (Pseudomonas phage), respectively. A K^+^ channel gene was also discovered in the genome of Streptomyces phage BillNye (123 aa, 2 TMs, accession #AVD99322.1) with some similarity to a hypothetical protein from *Pararheinheimera texasensis* (Gammaproteobacteria; blastp E value 1e−19, 34% aa identity, accession #WP_031568736.1). *Streptomyces* belongs to the *Actinobacteria* and is not related to *Pararheinheimera*. Hence, there is no direct evidence that a Steptomycete is a host for the channel carrying phage.

In addition, one K^+^ channel gene was found in *Lactobacillus* phage PLE (259 amino acids, four predicted TMs, accession #YP_009282368.1). The blastp search revealed that it is identical to a putative ion transporter from the *Lactobacillus casei* group, thus, a potential host of the phage (accession #WP_012491300.1).

The phylogenetic relationship of canonical K^+^ channels from viruses and phages (blue) and sequences from cellular organisms (red) that were most similar to the viral sequences are shown in Figure 2. The figure shows that K^+^ channels from different viruses show no obvious relationship. The relationship between the viral channel and non-viral K^+^ channels exhibit two different extremes. In one case the viral protein is identical to that of its host while in the other cases there is no apparent relationship between genes from virus/phages and their hosts.

Finally, it is worth noting that several proteins from viruses belonging to Mimiviridae are annotated as K^+^ channels (Table S4). They vary in size from 103 to 127 aa, contain two predicted TMs and show some similarity to bacterial K^+^ channels. However, they do not contain the crucial signature sequence of canonical K^+^ channels. Due to the functional importance of this domain for K^+^ channel function [40] we did not include these proteins in the current analysis.

The main novelty from the present analysis in relation to viral K^+^ channels is that they are not only present in viruses from algae but also in bacterial phages. The large diversity between the viral K^+^ channels argues against a common origin of the genes. A comparison between the viral K^+^ channels genes with those of the host does not provide an answer on their origin because we find genes, which are very similar but also very different from the respective host genes.

#### 3.1.2. Chloride Channels

Chloride channels consist of a larger and very heterogeneous family of proteins, which catalyze the passive diffusion of Cl^−^ across membranes. The proteins share little structural and functional features except that they all exhibit a rather low selectivity among anions [41].

**Figure 4:**
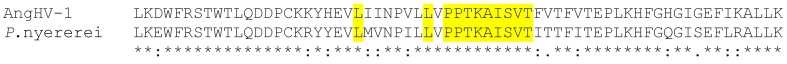
Partial sequence alignment of CLIC-like chloride channel proteins from AngHV-1 with a homolog in a fish. Alignments of the CLIC-like chloride channel with typical conserved regions (in yellow) from Anguillid herpesvirus-1 (AngHV-1) versus the chloride channel from the fish *Pundamilia nyererei* (*P. nyererei*). The complete alignment is shown in Figure S3A. In the alignment, identical amino acids are indicated by “*”, conserved and semi-conserved amino acids are indicated by “:” and “.”, respectively.

Some aquatic herpesviruses contain genes encoding two types of chloride channels: CLIC (chloride intracellular channel)-like chloride channels and MCLC (mid-1-related chloride channel)-like chloride channels. Anguillid herpes virus 1 (AngHV-1), which infects the eel *Anguilla anguilla,* carries a gene for a CLIC-like protein (281 aa, two predicted TMs, accession #YP_003358251). The most similar homologous proteins are from fish species, with a protein from *Pundamilia nyererei* being the most similar (blastp E value 7e−79, 67% aa identity; accession #XP_005746869.1) (Figure 4). No homologous sequence was found in the host genome possibly due to incomplete data. The homologous sequences from other organisms are much longer than the viral sequence (>530 aa) and contain at least one more predicted TM (Figure S3).

Abalone herpesvirus carries a gene for a MCLC-like protein (YP_006908742, 333 aa, three predicted TMs). Homologous sequences were found in the genomes of a variety of aquatic animals such as mussels, worms, fish and corals (Table S5) although with low similarity. The homologous sequences are of similar size or longer (265–685 aa) than the viral protein. Again there was no homolog in the host genome. The blastp search also revealed two sequences from Abalone herpes virus from two different locations and years of sequencing (YP_006908742.1 and AET44204.1) that differ in one aa (in position 304, Q and H, respectively) plus a partial sequence (ADL16677.1, 134 aa, identical to YP_006908742.1). Further, two more sequences were found from herpesviruses that infect other mussels (Chlamys acute necrobiotic virus, ADD24788.1 and Ostreid herpesvirus 1, YP_024600.1; 316 aa each). They differ in one aa in position 312, K and R, respectively.

The resume from a search on viral Cl^-^ channels is that there are different members of this family of anion channels in some DNA viruses. It is notable that they do not coexist with K^+^ channels suggesting that an efflux of KCl salt is not a functional feature.

#### 3.1.3. Ligand-Gated Ion Channels

Ligand-gated ion channels are activated by various molecules such as glycine and glutamate. They vary in size and structure. For example, human glutamate receptors are trimeric and are activated by glutamate, while glycine receptors are pentamers and are activated by glycine and other similar small aas [42].

**Figure 5:**
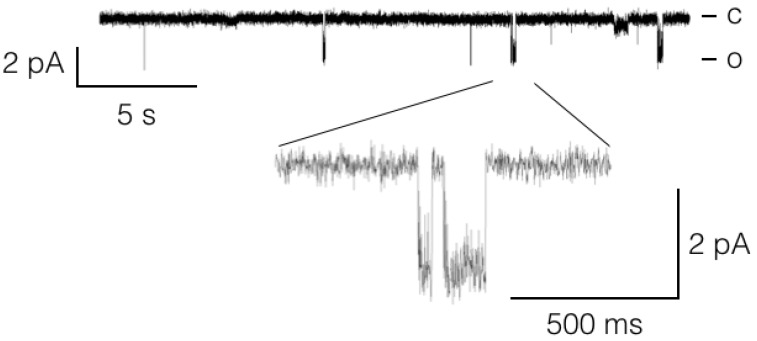
A chlorovirus glutamate receptor-like channel generates single channel fluctuations, which are elicited by 1 mM of glycine. Recordings were conducted with the proteins expressed in HEK293 cells and in cell-attached configuration with glycine in the pipette medium.

Chlorovirus PBCV-1 (*Phycodnaviridae*) and the closely related OSy-NE5 virus carry a gene for a putative glutamate receptor (411 aa, three TMs, NP_048510 and 406 aa, three TMs, YP_009325583.1, respectively; Figure S4A,B). No other viruses have a similar gene. The similarity to other glutamate receptors is small, however. The most similar protein is one annotated as ‘glutamate (NMDA) receptor subunit epsilon-2′ from *Cricetulus griseus* (E value 0.011, aa identity 22%; accession #ERE 66598.1). The viral protein is much smaller (411 aa vs. 1121 aa) and lacks a conserved n-methyl d-aspartate receptor 2B3 C-terminus domain, which is present in the mammalian homolog (Figure S4A,B; [42]). This large hydrophilic extra-cellular domain is typical of NMDA receptor proteins and presumably involved in receptor function. Apart from this domain the general architecture of the pore region in the viral protein slightly resembles a mammalian glutamate receptor (Figure S4B). Attempts to activate the channel after expression in *Xenopus* oocytes with glutamate failed. However when we measured channel activity in HEK293 cells expressing the protein we recorded single channel fluctuations with a conductance of ca. 20 pS. These fluctuations, examples are shown in Figure 5, were only seen in recordings when the pipette solution contained 1 mM of glycine (*n* = 5). In the absence of glycine (*n* = 5) or with 1 mM glutamate in the pipette (*n* = 5) these channel openings were not seen. This suggests that they originate from a glycine activation of the viral protein. An activation of this type of ligand activated channels is not completely surprising considering that channels with a similar architecture from plants are activated by glycine, but not by glutamate [43].

Several chlorovirus genomes contain a gene that is annotated as a ligand-gated ion channel, e.g., ORF A163L from PBCV-1 (433 aa, three predicted TMs, accession #NP_048511). The proteins are weakly similar to a hypothetical protein from the plant *Vigna angularis* (blastp E value 0.017, 24% aa identity, accession #KOM43022.1) and a “glutamate receptor 2.8-like” protein from the plant *Vigna radiata* var. *radiata* (blastp E value 0.025, 25% aa identity, accession #XP_014499575.1). There is no indication that these two plants are hosts for these viruses.

The interesting conclusion, which can be drawn from a screening of viral genomes for ligand gated channels, is that distantly related members of this family of channels are present in a few viral genomes. The genes products seem to be functional and indeed gated by ligands. The physiological function of the viral channels remains unknown. However, their low abundance in viral genomes suggests that they may not be as essential as K^+^ channels.

#### 3.1.4. Aquaglyceroporins

Aquaglyceroporins are a subgroup of the aquaporin family of membrane proteins. They augment the diffusion of water, glycerol and other small, non-ionic molecules across membranes. In addition to their role in osmoregulation they have many other physiological functions in all forms of life [44]. Several, but not all, chloroviruses in the SAG and Pbi virus groups carry a gene coding for an aquaglyceroporin (e.g., AQPV1_MT325_ 270 aa, six predicted TMs, accession #ABT13584). Characterization of the protein has been described elsewhere [18]. A similar gene has been found in the recently described chlorovirus OSY-NE5 that belongs to a separate group of chloroviruses (270 aa, six predicted TMs, accession #YP_009325532.1) that infect a different chlorella host. Currently there is no evidence that these genes originated from their current hosts.

#### 3.1.5. Mechanosensitive Channels

Mechanosensitive channels are predominantly present in bacteria but they also exist in eukaryotes where they facilitate a rather nonselective flux of ions. The name refers to their type of gating; they are activated by mechanical changes to the membrane [45].

Aureococcus anophagefferens virus has a gene coding for a 261-aa long putative mechanosensitive channel that contains three predicted TMs (accession #YP_009052121.1). It is most similar to a “mechanosensitive ion channel protein MscS” from *Nesiotobacter exalbescen*s. However, the similarity is low (blastp E value 8e−10, 25% aa identity, accession number WP_028481655.1) and the bacterial protein is much larger (843 aa vs. 261 aa).

An even smaller putative mechanosensitive channel was detected in Tetraselmis virus 1. The 101 aa long protein contains one to two predicted TMs (accession #AUF82136.1) and is most similar to a ‘large conductance mechanosensitive channel protein’ from a bacterium in the Parcubacteria group. Again, the similarity is rather low (blastp E value 4e−5, 29% aa identity, accession #KKP92070.1), but the proteins are similar in size (127 aa vs. 101 aa).

A protein of the same size was also detected in the genome of the Cafeteria roenbergensis virus BV-PW-1 (accession #YP_003969926.1). It has two predicted TMs and has low similarity to a putative large-conductance mechanosensitive channel from an Arc I group achaeon (blastp E value 1e−4, 27% aa identity, accession #KYC46208.1). Interestingly, the three viral proteins are not similar to each other (Figure S5). This suggests at least three independent transfers of mechanosensitive ion channel genes into a viral genome; however, evidence for a function for these proteins is still missing. In addition, the low similarities to proteins from cellular organisms do not provide much information on a potential origin of the genes.

Our databank screening confirms that different types of mechanosensitive channels are present in viral genomes. This includes the large mimiviruses but also the much smaller Tetraselmis virus, which has an alga as host. The question as to whether the putative channels are active and whether they play a role in viral replication remains unanswered.

#### 3.1.6. Other Ion Channels

Another type of ion channel is the Golgi anti-apoptotic protein (GAAP). These proteins are part of the TM Bax inhibitor-containing motif family (TMBIM) and they modulate Ca^2+^ signaling [46]. Several poxviruses, including camelpox virus, cowpox virus and vaccinia virus, have a gene coding for a putative GAAP that is very similar to proteins from mammals (Tables S6 and S7). A study has reported that the viral GAAP is functional, i.e., it is anti-apoptotic and modulates intracellular Ca^2+^ [47]. In contrast to the human homolog, the viral protein functions as a monomer.

### 3.2. ATPases and ABC Transporters

While the aforementioned channels catalyze a passive transport of ions, ATPases are large enzymes, which hydrolyze ATP for an active transport of substrates against their concentration gradient across membranes [48]. They are found in all domains of life including viruses [19]. The main classes of ATPases, which differ in their structure and function, comprise the F- (or V-) and the P-Type ATPase. The so-called ABC transporters can also be classified in this group [48].

#### 3.2.1. ATPases

The Ca^2+^ transporting ATPases of Pbi chloroviruses and other chloroviruses have been functionally characterized and are described elsewhere (Table S1; [19]). In addition, the more recently discovered Syngen chlorovirus OSy-NE5 contains a very similar gene (blastp E value 0.0, 67% aa identity; accession #YP_009325856.1).

Putative Mg^2+^ transporting ATPase encoding genes are present in some of the recently discovered large viruses, such as Klosneuvirus [49]. The putative gene product of the Klosneuvirus-1 gene has a length of 922 aa and contains ten predicted TMs (accession #ARF11485.1). The most similar protein is a “phospholipid-translocating P-type ATPase” from *Trichomonas vaginalis* G3 (blastp E value 2e−46, 25% aa identity, accession #XP_001311486.1). Homologous genes were also detected in Tupanvirus from a soda lake (accession #AUL77738.1), Tupanvirus from the deep ocean (accession #AUL79024.1) and Hokovirus-1 (accession #ARF10438.1).

Catovirus-1 has a gene coding for a putative P-type ATPase. The protein has a length of 809 aa and has six to seven predicted TMs (accession #ARF08724.1). This is similar to P1A-type ATPases that are involved in K^+^ transport (accession #ARF08724.1). The most similar protein is a “phospholipid-translocating P-type ATPase” from *Trichomonas vaginalis* G3. However, it is a slightly different protein from the one described above (blastp E value 5e−21, 22% aa identity, accession #XP_001330565.1). Both proteins contain the conserved phosphorylation domain DKTGT, but otherwise they differ from each other (see alignment in Figure S6).

The search for ATPases in viral genomes shows that they are indeed present in large DNA viruses. If additional studies confirm their activity the data imply that viral proteins are able to facilitate active ion transport and with that generate electrochemical gradients across membranes.

#### 3.2.2. ABC Transporters

ABC transporters (ATP-binding cassette transporters) are large proteins that utilize ATP in order to transport substrates across membranes. They are found in all cellular organisms and transport various substrates including complex organic molecules in and out of cells [48].

**Figure 6:**
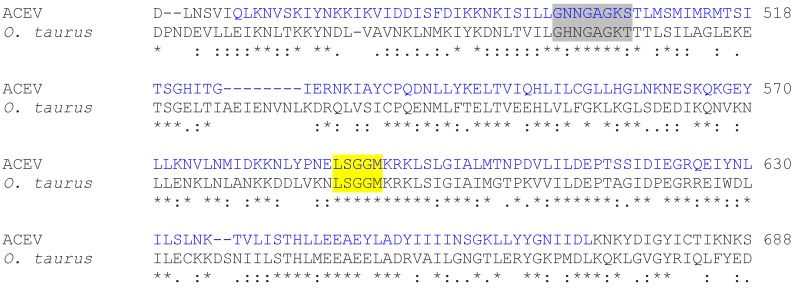
Partial sequence alignment of ATP-binding casette (ABC)—transporter type protein from virus ACEV and the most similar protein from a beetle. Alignment of insect virus ACEV versus the homologous protein from the beetle *Onthophagus taurus*. The highly conserved Walker A motif (in grey) and the conserved LSGGQ(LM) motive (yellow) are embedded in conserved ATP-binding cassette domains (blue). The complete alignment is shown in Figure S7. In the alignment, identical amino acids are indicated by “*”, conserved and semi-conserved amino acids are indicated by “:” and “.”, respectively.

The insect viruses Anomala cuprea entomopoxvirus (ACEV) and Amsacta moorei entomopoxvirus (AMEV) are the only viruses that carry a gene coding for a large ABC transporter. In the ACEV genome ORF ACV025 is the largest ORF coding for a protein of 1506 aa ([50]; accession #YP_009001498.1). The most similar proteins are from beetles (e.g., the ATP-binding cassette sub-family A member 3-like isoform X1 from *Onthophagus taurus*, blastp E value 0; 34% aa identity; XP_022911124.1); the high degree of similarity suggests that the gene was only recently derived from the host (Figure 6). The viral protein is slightly shorter than the insect homolog (1506 vs. 1571 aa), but contains the structural elements of an ATP-binding cassette transporter, i.e., TM domains and the nucleotide binding domain (NBD) with two Walker A motifs (GXXGXGKS/T) in the order TMD-NBD-TMD-NBD (Figure S7). Also a known variant of the conserved LSGGQ motive from ABC transporters [48] is present as LSGGM in both proteins. The partial alignment of the viral and beetle proteins highlights the position of one of the two Walker motives in a conserved ATP-binding cassette (Figure 6).

Interestingly, the two viral proteins are not very similar (blastp E value 2e−43, 31% aa identity; NP_064912.1) and the 1384 aa protein from AMEV is smaller in size, but still has the same overall structure of an ABC transporter. The latter protein is most similar to proteins from butterflies (e.g., *Helicoverpa armigera*, blastp E value 3e−169, 24% aa identity; XP_021196765.1). *Amsacta moorei* is also a butterfly.

ABC transporter genes are also present in the genomes of several mimiviruses (e.g., Mimivirus, Mamavirus, Moumouvirus) and related viruses, including Tupanvirus, Catovirus, Indivirus, organic lake phycodnavirus, and Pithovirus sibericum. The viral ABC transporters exhibit high diversity. They differ in size from 452 to 605 aa and contain one to five predicted TMs (Table S8). They all contain the highly conserved Walker A motif, but only one copy, in contrast to the entomopoxvirus ABC transporters.

**Figure 7:**
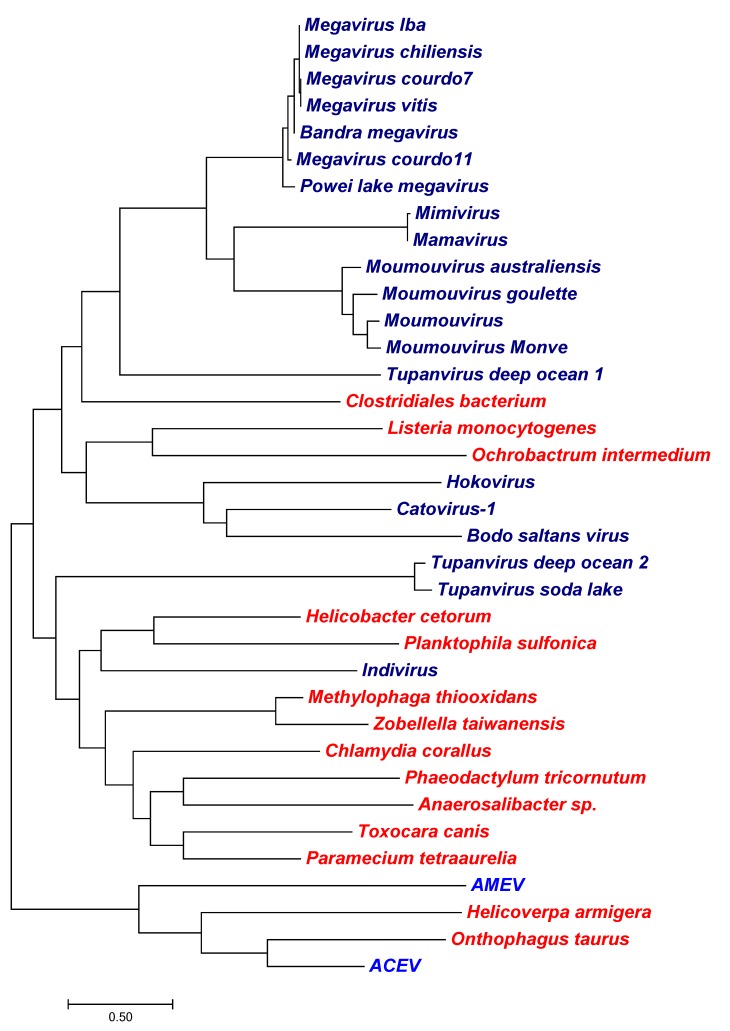
Maximum likelihood tree of ATP-binding casette (ABC)-transporters. The tree was constructed as in Figure 2 and includes sequences from viruses (blue) and sequences from cellular organisms (red) that were most similar to the viral sequences. Shown is the tree with the highest log likelihood (−17,822.82). The analysis is based on 36 protein sequences with 272 positions in the final dataset.

The phylogenetic tree in Figure 7 shows the relationship between the viral ABC transporters and the most similar proteins from cellular organisms. The tree implies different origins for the viral proteins. For example, the Indivirus protein clusters within the group of prokaryotic and eukaryotic ABC transporters and it is separated from the majority of giant virus proteins. Interestingly, Tupanvirus from the deep ocean has two different ABC transporters. One that is closely related to the respective protein from Tupanvirus from a soda lake, and one that forms a sister clade to the other giant virus ABC transporters.

The screening of viral genomes for ABC transporters reveals an unexpected diversity of members of this family in a wide range of different types of DNA viruses. With no functional data available it is impossible to speculate on any functional relevance of these proteins. Additionally, the phylogenetic data provide no clear-cut clue on the origin of these genes in viruses. The apparent sequence diversity of the viral genes however argues against a common ancestor for these genes.

### 3.3. Transporters

In addition to channels and ATPases, cell membranes also contain a large scope of additional transport proteins with different functions and structures. Unlike ATPases these transporters do not perform active transport. They are distinguished from channels on the basis of their turnover rates, which is lower than that of channels. The role of these transport proteins is to facilitate movement of charged and uncharged molecules across membranes. They can do this by facilitating the diffusion of the molecules down an (electro)-chemical gradient or by a secondary active process. In the latter case the molecule of interest (e.g., glucose, amino acids) is transported in a symport or antiport manner. In both cases a second molecule is moving down its electrochemical gradient to transport the solute of interest up hill. These transporters are often classified with respect to the primary solute that they transport [51]. In the following sections we describe a number of viral genes for different types of transporters with specific substrate preferences. Currently there are only a few reports on the functionality of these transporters and even less information on their physiological role.

#### 3.3.1. Potassium Transporters

Genes encoding the potassium transporters of the HAK/KUP/KT type are present in two families of chloroviruses. They are functional and have been described elsewhere (see Table S1; [20]). Recent plastid genome sequencing of one of the hosts, *Micractinium conductrix*, [52] revealed a putative potassium transporter protein with high similarity to the potassium transporter HAKCV from chlorovirus Fr483 (blastp E value 2e−137, 39% aa identity, accession #PSC73584.1). The host protein is much longer (1343 aa vs. 660 aa) and has a large C-terminus (Figure S8). The same is true for the homologous protein from the related green alga *Chlorella sorokiniana* (1239 aa; accession # PRW58025).

#### 3.3.2. Magnesium Transporters

Magnesium transporters comprise a large and heterogeneous family of proteins, which catalyze the passive and active transport of Mg^2+^ across membranes [53]. Two *Lactobacillus* phages each have one gene encoding a putative magnesium transporter: Lactobacillus phage Lfelnf (322 aa, two or three predicted TMs, accession #YP_009222254.1) and Lactobacillus virus Lb338-1 (329 aa, two or three predicted TMs, accession #YP_002790748.1). They are very similar to each other (blastp E value 0.0, 87% aa identity) and somewhat similar to a putative magnesium transporter from *Lactobacillus hokkaidonensis* (blastp E values and aa identities were 1e−34 and 32% (Lactobacillus phage Lfelnf) and 2e−33 and 31% (Lactobacillus virus Lb338-1); accession #WP_041093908.1; Figure S9). The topology prediction algorithm indicated two TM domains for both the viral and the bacterial protein. However, experimental results indicate that the bacterial protein contains three TMs [54].

#### 3.3.3. Ammonium Transporters

**Figure 8:**
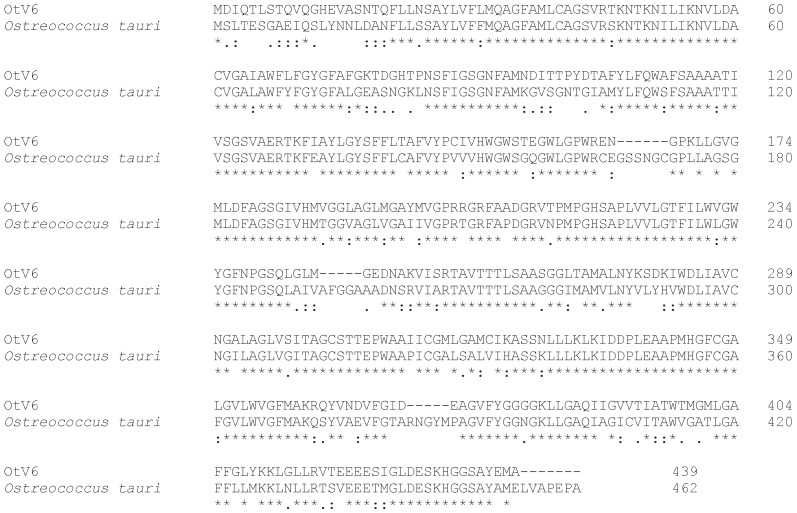
Sequences of ammonium transporter from virus and host are very similar. Alignment of ammonium transporter from virus OtV6 and its host *Ostreococcus tauri*. Note the high degree of homology between the two sequences. In the alignment, identical amino acids are indicated by “*”, conserved and semi-conserved amino acids are indicated by “:” and “.”, respectively.

Ostreococcus tauri virus (OtV6) has a gene coding for a functional ammonium transporter (439 aa, 11 predicted TMs; accession #AFC35023.1; [55]). It is most similar to a protein from its host *Ostreococcus tauri* (blastp E value 0.0, 71% aa identity; accession #XP_022840606.1 (Figure 8). A second putative ammonium transporter gene has been detected in Chrysochromulina ericinia virus. The protein has a length of 124 aa and has two predicted TMs (accession #YP_009173407.1). It slightly resembles an ammonium transporter protein from *Lysinibacillus xylanilyticus* (blastp E value 9e−13, 41% aa identity, accession #WP_049666291.1). Interestingly, the viral protein is longer than its bacterial homologs (124 vs. 108/109 aa).

#### 3.3.4. Sodium/Phosphate Symporters

**Figure 9:**
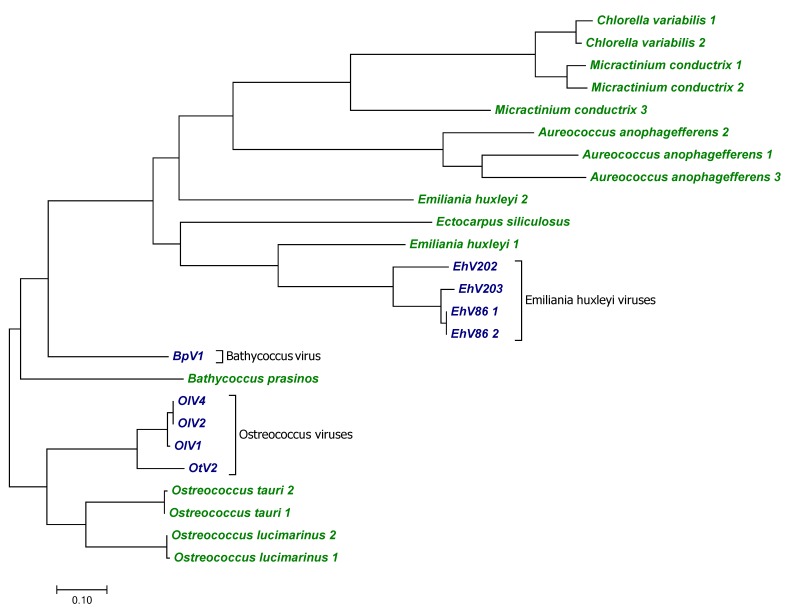
Phylogenetic comparison between viral and host sodium phosphate transporters. The tree was constructed as in Figure 2 and includes sequences from aquatic viruses (blue) and their hosts (green). Shown is the tree with the highest log likelihood (−5038.51). The analysis is based on 25 protein sequences with 193 positions in the final dataset.

The marine coccolithoviruses infecting *Emiliania huxleyi* (EhV86, EhV202 and EhV203) and the prasinoviruses infecting *Bathycoccus* (BpV1) and *Ostreococcus* species (OlV1, OlV2, OlV4, and OtV2) each carry a gene coding for a putative sodium/phosphate symporter (Table S9). The proteins have a length of 459 to 534 aa and contain 9 or 10 predicted TMs. A blastp search shows that the viral genes are similar to phosphate transporters from a fish and various alga species. However, given that most of the hits were sequences from algae and especially from hosts or host-related species, we suspect that the fish sequence is the result of contamination. For example, the blastp search for the protein from EhV84 (accession #AEO97646.1) revealed a “phosphate-repressible phosphate permease” from *Emiliania huxleyi* (blastp E value 7e−162, 60% aa identity, accession #AAO15381.1). The phylogenetic tree in Figure 9 shows that the viral genes are more similar to genes from their respective hosts than to each other. These results suggest at least three independent events of sodium/phosphate transporter gene transfers in these viruses.

#### 3.3.5. Sodium/Glucose Transporters

Three Aeromonas phages carry a gene coding for a putative sodium/glucose co-transporter: Aeromonas virus 44RR2 (NP_932589.1), Aeromonas virus 31 (YP_238960.1) and Aeromonas phage Riv-10 (APU02286.1). All three proteins have a size of 507 aa and contain 14 predicted TMs. They share the overall topology of known sodium/glucose transporters (Figure S10). The sequences are very similar to bacterial proteins. For example, the protein from Aeromonas virus 44RR2 was most similar to a “sodium/glucose cotransporter” from *Photobacterium sanctipauli* (blastp E value 3e−145, 48% aa identity, accession #WP_107271833.1). The host of the Aeromonas phages is *Aeromonas salmonicida* that itself is a fish pathogen. It is unknown if the phages infect other bacteria, such as *Photobacterium*.

#### 3.3.6. Sodium/Calcium Symporter

Two phages carry a gene coding for a putative sodium/calcium symporter: Vibrio phage 1.084.O. 10N.261.49.F5 (370 aa, nine predicted TMs, accession #AUR86274.1) and Pseudoalteromonas phage J2-1 (377 aa, nine predicted TMs, accession #ATN93525.1). They are similar to bacterial proteins, e.g., the Vibrio phage protein is similar to a sodium/calcium symporter from *Vibrio jasicida* (blastp E value 2e−74, 40% aa identity, accession #WP_105067875). The similarity between phage and host genes suggests a close relationship (Figure S11).

#### 3.3.7. Nicotinamide Mononucleotide Transporters

**Figure 10:**
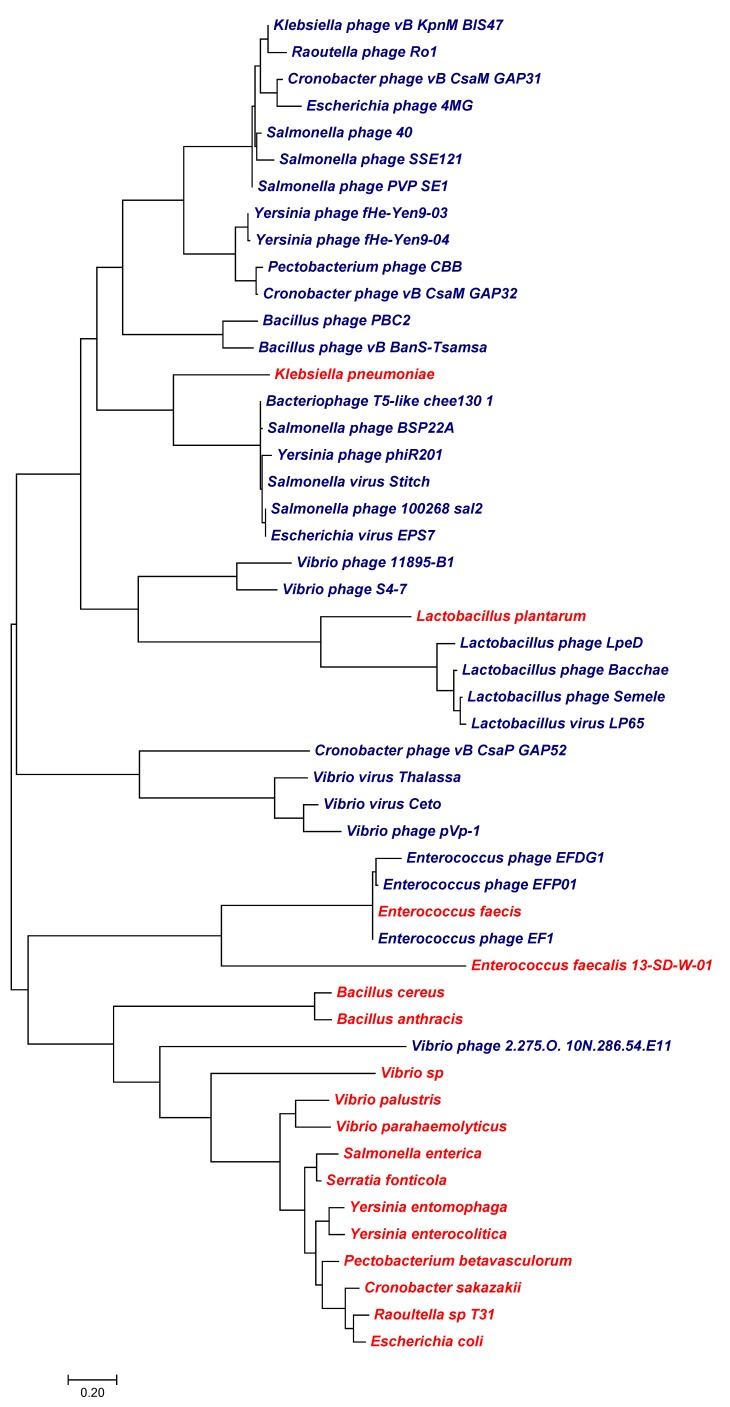
Phylogeny of nicotinamide mononucleotide transporters. Maximum likelihood tree of phage nicotinamide mononucleotide transporters (blue) and protein sequences from hosts or host-related bacteria species (red). The tree was constructed as in Figure 2. Shown is the tree with the highest log likelihood (−7219.01). The analysis is based on 50 p sequences with 142 positions in the final dataset.

At least 35 phages infecting various bacteria species have a gene for a nicotinamide mononucleotide transporter (Table S10). The proteins vary in size from 225 to 267 aa and contain six to eight predicted TMs. Figure 10 shows a phylogenetic tree of viral sequences as well as sequences from their hosts; in those cases in which the host genomes were not available sequences from a member in the genus were used. The tree revealed a similarity between viral and bacterial proteins. For example, the protein from a Vibrio phage is on the same branch with bacteria from the Vibrio genus, and the proteins from the Lactobacillus phages are on the same branch as a protein from a *Lactobacillus* species. Additionally, the protein from Enterococcus phage EF1 is identical to “nicotinamide riboside transporter” from *Enterococcus faecium* (blastp E value 0.0, 100% aa identity, accession #WP_073981706.1). The results of this analysis suggest a recent gene transfer between host and virus.

#### 3.3.8. Nucleoside Transporters

Two herpesviruses carry a gene coding for a putative nucleoside transporter: Harp seal herpesvirus and F22 (Felis catus gammaherpesvirus 1). The proteins have a length of 432 aa (Harp seal herpesvirus; AJG42984.1) and 429 aa (Felis catus herpesvirus 1; YP_009173407.1), respectively, and contain 11 putative TM domains. A BLAST search shows a high similarity to mammal nucleoside transporter sequences, e.g., to “nucleoside transporter 1” from *Desmodus rotundus* (blastp E value 3e−118, 43% aa identity, NCBI accession #XP_024413597.1).

#### 3.3.9. Amino Acid Transporters

Amino acid permeases are relatively large proteins that facilitate the transport of various aa. They belong to the solute carrier family and they usually have 12 TMs [56]. Several fish-infecting iridoviruses have a gene coding for a putative aa permease (e.g., Red seabream iridovirus, 378 aa, 10/11 TMs, accession #BAK14282). There are five viral sequences with small aa changes and one with many differences. The closest non-viral homologs are proteins from *Xenopus tropicalis* (blastp E value 8e−16, 26% aa identity, XP_017947765.1) and from *Pagrus major* (Red seabream; blastp E value 1e−15, 25% aa identity, XP_015484305.1). The similarity is low, though, and the viral proteins are shorter (see alignment in Figure S12).

### 3.4. Other Proteins

Table S11 shows more examples of channel and transporter encoding genes found in phages. The sequences are identical to sequences of bacterial genes and mostly to genes from hosts or host-related bacteria.

#### Putative Transport Proteins

Most large dsDNA viruses have several uncharacterized genes coding for putative membrane proteins. Without experimental data we can only speculate on their transport function. There are, however, some data about virion-associated proteins from chloroviruses, which suggest that some of these proteins may be involved in membrane conductance. PBCV-1 and other chloroviruses from different families have at least three genes that code for putative membrane proteins:

(i) ORF A201L (accession #AAC96569.1) codes for a 94 aa protein with two predicted TMs. It has weak similarity to proteins from Archeae.
(ii) ORF A621L (accession #AAC96952.2) codes for a 117 aa protein with two TMs and weak similarity to the potassium channel Kcv. There are no non-viral homologs so far in the databases.
(iii) ORF A624R (accession #AAC96955.1) codes for a 121 aa protein with two or three predicted TMs. It also contains the DUF2177 conserved domain that is so far not characterized.

**Figure 11:**
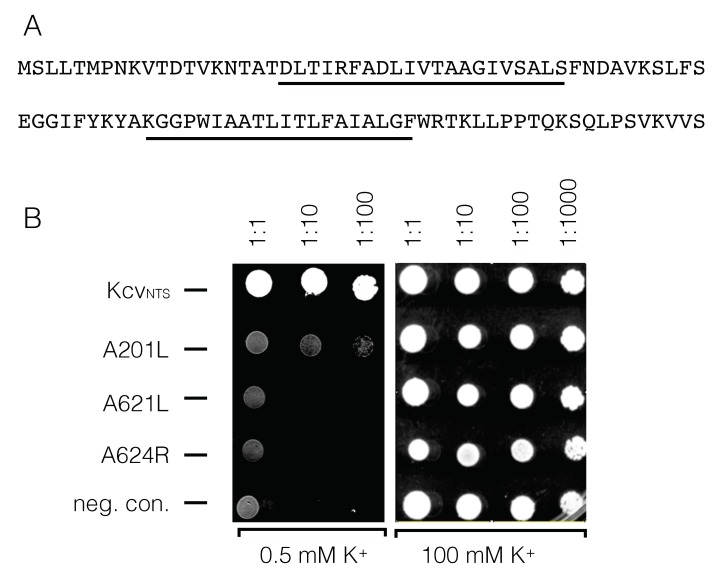
Growth phenotype of ΔtrkΔtrk2 yeast mutants transformed with putative membrane proteins from different chloroviruses. (**A**) Amino acid sequence of open reading frame A201L from virus NY-2A. Predicted transmembrane domains are underlined. (**B**) Yeast cells were transformed with positive control (Kcv_NTS_), open reading frames A201L, A621L, and A624R from NY-2A or negative control (empty vector). All yeasts were plotted at different dilutions: 1:1 (undiluted), 1:10, 1:100, and 1:1000 on selective medium containing 0.5 mM K^+^ or on non-selective medium with 100 mM K^+^. Yeast transformed with A201L from virus NY2A partially rescues yeast growth on selective medium with low K^+^ concentrations.

To test for a putative channel function of these proteins we expressed them in yeast mutants, which lack a functional K^+^ uptake system. The experiments are based on the assumption that a rescue of yeast growth on a selective medium with low K^+^ provides indirect evidence for a channel function for the viral protein [57]. Experimentally, we found that only the homologous protein to ORF A201L from chlorovirus NY-2A (accession #YP_001497468.1) was able to rescue the yeast mutant. The results of these experiments (Figure 11) suggest that neither ORF A621L nor ORF A624R have any K^+^ channel activity. The positive rescue of growth by ORF A201L on the other hand can be interpreted as evidence for a channel function for this protein. However, the electrophysiological characterization in HEK293 cells did not result in a robust K^+^ channel function.

The general message from this analysis is that viral genomes contain a large number of genes, which code for membrane proteins with no apparent homologs in pro- or eukaryotes. The data suggest that some of these gene products may have a transport function. A functional analysis of these proteins may uncover novel structure/function correlates in transport proteins and presumably more insights into the functional roles of membrane proteins in virus infection and/or replication.

## 4. Discussion

A key message from this study is that viral genes encoding membrane transport proteins are not as rare in viruses as might be expected. The databank search shows that homologs to all major classes of membrane transport proteins that are present in prokaryotic and eukaryotic organisms are also encoded by some viruses. This includes genes for channels with different ion selectivities and modes of activation, transporters for various charged and non-charged molecules, as well as for ATPases. In addition to these proteins with apparent homology to non-viral proteins there are many more putative viral genes encoding membrane proteins with unique structures. An increasing number of studies over the past few years have shown that many of these viral structural proteins exhibit transport functions [2,3,4,5]. Since the latter are only discovered in the context of functional studies and not with bioinformatics tools the real number of unique viral transport proteins is probably underestimated.

### 4.1. Abundance of Viral Transport Proteins and Function

It is generally believed that viruses only contain genes if a gene product provides a benefit at some stage of infection/replication or under specific environmental conditions. Thus far only a few viral encoded transport proteins have been examined with respect to their function. This includes a group of proteins with homology to prokaryotic and eukaryotic K^+^ channels [12], K^+^ transporters [20], an aquaglyceroporin [18], a Ca^2+^ transporting ATPase [19], as well as the GAAPs of the poxviruses [46,47]. The data presented in Figure 5 suggest that the putative ligand-gated channel-like protein from a chlorovirus could also be functional. Additionally, within this group of unique viral transport proteins, function was confirmed by several assays [3,4,5,58]. The general message from these experiments is that the majority of viral transport proteins that have been tested are functional. However, this information does not provide any clues as to their physiological role(s). For many of the viral channels and transporters there is circumstantial evidence for a role of ion conducting properties in virus entry, replication, particle assembly, budding, or induction of apoptosis in host cells [5,58]. The fact that many viral proteins, however, exhibit dual functions [58,59] makes a clear-cut understanding of the relationship between ion conductance and physiological function difficult.

In the case of the Vpu protein from HIV-1, which has conserved its ion conducting property through evolution [10], it is still unknown if the channel property of the protein is important [60,61]. In a few selected cases such as the M2 protein from influenza virus A and the K^+^ channels in viruses infecting Chlorella-like green algae it is possible to relate the function of the viral protein with distinct steps in the infection or replication of their respective viruses [15,16,17,62]. The physiological role of the remaining viral transport proteins with either confirmed function or with unknown function is unknown. From the current data it seems as if viral membrane transport proteins with homologs in eukaryotes and prokaryotes are most often present in the genomes of large DNA viruses and often present in aquatic viruses. It is noticeable that many of the proteins have been found in the genomes of phycodnaviruses and therein, especially in the genomes of chloroviruses.

Some clues on the functional importance of a transporter may be derived from the frequency with which the gene is present in a group of viruses. In this context it is important to note that most chloroviruses have a K^+^ channel gene. This finding is in good agreement with the experimental data, which demonstrate the importance of the viral K^+^ channel in the infection of the host and in a mutual competition between different chloroviruses by preventing hyperinfection [16]. K^+^ channel genes are also common in phycodnaviruses infecting unicellular marine algae [39] and the filamentous brown alga *Ectocarpus siliculosus* [63]. We assume that they also play an important physiological role in these viruses. However, at this point we cannot pinpoint their exact function in these virus/host systems. We do know that it must differ from that in the chlorovirus/host system, at least for the Ectocarpus siliculosus virus. This virus does not infect intact cells with a cell wall but infects wall-less gametes or spores of their algal hosts [64]. This fundamental difference in the infection pathway does not require a role for the EsV1 Kesv channel in reducing the K^+^ content in the host cell as occurs in *Chlorella* cells. This conclusion agrees with other data, which show that in heterologous expression systems the Kesv channel is not sorted to the plasma membrane of cells but to the mitochondria [63]. The physiological significance of this finding is unknown but it implies a fundamentally different function for the channel in this marine virus.

The present data indicate that K^+^ channel encoding genes are also common in vibriophages. This again implies that the proteins have a role in the phage infection/replication cycles that is not required in other phages, which lack these genes. At this point any suggestion for a function is speculative. But it is interesting to note that other phages like T4 or T5, which infect Gram-negative bacteria, need to augment the K^+^ conductance of their host as a prerequisite for DNA injection into the host [65,66]. To determine if the viral K^+^ channels from vibriophages are involved in such a process needs to be tested.

While K^+^ channel proteins are common in some viruses they only occur sporadically in others. Using the argument that only the abundant occurrence of a gene indicates a basic function for its product we suggest that some K^+^ channels are not essential in viruses, like in the mycobacterium phage Myrna.

In contrast, as identified here ATP type ABC transporter encoding genes are present in many mimiviruses and related viruses. Because of the general abundance in this group of viruses we suggest that they play an important role in the viral life cycle.

A third gene that is common in many phages codes for nucleotide transporters. We suspect they play an essential role in the phage life cycles because they are so widespread.

Using the abundance argument it is worth noting that some, but not all, chloroviruses encode genes for other transport proteins in addition to the K^+^ channel protein [14]. The SAG virus ATCV-1, for example, has, in addition to a gene for a K^+^ channel protein, genes encoding a K^+^ transporter, an ABC transporter, an aquaglyceroporin, and at least three additional putative transport proteins (homologous to ORFs A201L, A621L and A624R from PBCV-1). The fact that some of these genes are not present in all chloroviruses suggests that they may not serve an essential role in the viral infection/replication cycles, or else they have a backup system to compensate for the missing gene(s). Since the chloroviruses are present throughout the globe [67,68], it might be that the additional transporters provide a benefit under specific environmental conditions.

### 4.2. The Evolutionary Origin of Viral Transport Proteins

The present data provide some interesting new insights into the possible origin of viral membrane transporters genes. A previous study reported that K^+^ channels from chloroviruses and from the Ectocarpus virus EsV1 are more similar to each other than to the homologs from their hosts [24]. This led to the conclusion that the viral channels have a long common evolutionary history and that they were probably not acquired from their present hosts [25]. It was further speculated that the viral channels might even have been the evolutionary origin of all K^+^ channels. The present data provide some new information about this subject. The data show that genes with the hallmarks of canonical K^+^ channels are not restricted to members of Phycodnaviridae, but that they are also present in the genome of a mimivirus and in some phage genomes. Direct comparisons of the viral K^+^ channels indicate that they are conserved within the different viral groups. However, they differ in size and primary aa sequence between different groups (Figure 2). This argues against a common origin for all viral K^+^ channels. The most interesting information comes from a comparison between K^+^ channels from members of Phycodnaviridae versus the channel from the recently characterized TeV-1. Both viruses belong to the nucleo-cytoplasmic large DNA viruses (NCLDV) and share a common set of core genes. Still the K^+^ channel genes from the mimiviruses and the phycodnaviruses are very different suggesting that the K^+^ channel genes are unlikely to originate from the same source. Taken together, these considerations suggest that the K^+^ channel genes were acquired multiple times by viruses. However, the available data in gene banks do not provide any indication of the origin of either of these genes. None of the genes have a similar homolog in their current hosts.

In other cases the origin of viral transport proteins is more obvious. Extreme cases are the genes shown in Table S11, which are identical to host protein sequences. We assume that the genes were only recently acquired from the respective hosts via molecular piracy. Many other proteins show at least small differences from eukaryotic or prokaryotic homologs, such as the poxvirus GAAPs or the Aeromonas phage sodium/glucose transporters. These genes were most likely acquired from their host cells. In the case of the two ATP-binding cassette transporters from the entomopoxviruses the gene products are more similar to the respective host family proteins than to each other. This suggests an example of two recent and independent gene transfers from the host to the virus; this large type of transporter may be important at some stage in viral replication.

## Supplementary Materials

The following supplementary materials are available at http://www.mdpi.com/1999-4915/10/9/456/s1. Table S1. Viral membrane transport proteins with homologs in living organisms. Table S2. Viral membrane transport proteins found in the databases. Table S3. Putative K^+^ channel proteins from vibrio phages. Table S4. Putative K^+^ channel-like proteins from large DNA viruses. Table S5. Blastp results of the MCLC-like protein from Abalone herpesvirus. Table S6. Results of blastp search of camel poxvirus GAAP against viral protein sequences. Table S7. Blastp results of camel poxvirus GAAP versus living organisms. Table S8. ABC transporter diversity among large DNA viruses. Table S9. Putative sodium phosphate transporters from viruses that infect marine picoplankton. Table S10. Phage nicotinamide mononucleotide transporter variety. Table S11. Phage channels and transporters that are identical Figure S1. Alignments of virus potassium channels. Figure S2. Putative K^+^ channels from vibrio phage. Figure S3. Putative CLC-like chloride channels from AngHV-1. Figure S4. Putative glutamate receptor-like channels from *Phycodnaviridae*. Figure S5. Putative mechanosensitive-like channels from large DNA viruses. Figure S6. Giant virus ATPases. Figure S7. Comparison of the ABC-transporter protein from virus ACEV and the most similar protein from beetle. Figure S8. Comparison of viral K^+^ transporter from virus and its host. Figure S9. Sequence and topology of Lactobacillus phage magnesium transporter. Figure S10. Sequence and topology of the Aeromonas virus 44RR2 sodium/glusose transporter. Figure S11. Comparison of a viral sodium/calcium symporter with the most similar protein from a Vibrio bacterium. Figure S12. Comparison of viral aa transporter with the most similar protein from a fish.
