# Supplementary material for: Genes for Membrane Transport Proteins: Not So Rare in Viruses
Source: Viruses. 2018 Aug 26;10(9):456. doi: 10.3390/v10090456 (PMC6163359; doi:10.3390/v10090456)
Supplement: Supplementary file 1 [file viruses-10-00456-s001.pdf]

Supplementary Material

Table S1. Viral membrane transport proteins with homologs in living organisms**.** The shown proteins have been functionally characterized. Alga species are indicated by *.

Protein	NCBI Accession #	Length in aa, (Predicted TMs)	Virus	Virus family	Genome size in base pairs, (accession #)	Host (Phylum)	Reference
NC64A chlorovirus potassium channel Kcv	NP_048599.1	94 (2)	Paramecium bursaria chlorella virus-1 (PBCV-1)	Phycodnaviridae (Genus chlorellavirus)	330.661 (JF411744.1)	*Chlorella variabilis* NC64A* (formerly: *Chlorella* NC64A) (Green algae)	Plugge et al., 1999
Pbi chlorovirus potassium channel Kcv	ABA40764.1	95 (2)	Chlorella Pbi virus MT325	Phycodnaviridae (Genus chlorellavirus)	314.335 (DQ491001.1)	*Micractinium conductrix** (formerly: *Chlorella* Pbi) (Green algae)	Gazzarrini et al., 2006
SAG chlorovirus potassium channel Kcv	YP_001427066.1	82 (2)	Acanthocystis turfacea chlorella virus-1 (ATCV-1)	Phycodnaviridae (Genus chlorellavirus)	288.047 (NC_008724.1)	*Chlorella heliozoae** (fomerly: Chlorella SAG3.83) (Green algae)	Gazzarrini et al., 2009
Prasinovirus potassium channel KBpV	YP_004061440.1	83 (2)	Bathycoccus sp. RCC1105 virus (BpV1)	Phycodnaviridae (Genus Prasinovirus)	198.519 (NC_014765.1)	*Bathycoccus* sp. RCC1105* (Green algae)	Siotto et al., 2014
Prasinovirus potassium channel KMpV	YP_004062056.1	79 (2)	Micromonas sp. RCC1109 virus (MpV1)	Phycodnaviridae (Genus Prasinovirus)	184.095 (NC_014767.1)	*Micromonas sp.* RC1109* (green algae)	Siotto et al., 2014
Prasinovirus potassium channel KotV	AFC34969.1	104 (2)	Ostreococcus tauri virus RT-2011 (OtV6)	Phycodnaviridae (Genus Prasinovirus)	189.567 (JN225873.1)	*Ostreococcus tauri** (Green algae)	Siotto et al., 2014
Phycodnavirus potassium channel KOLPV	ADX06199.1	140 (2)	Organic Lake phycodna virus 2 (OLPV-2)	Phycodnaviridae (environmental sample)	282.077 (HQ704803.1)	Unknown alga*	Siotto et al., 2014

Aureococcus virus potassium channel KAaV	YP_009052228.1	156 (2)	Aureococcus anophagefferens virus isolate BtV-01 (AaV)	Phycodnaviridae	370.920 (NC_024697.1)	*Aureococcus anophagefferens** (Heterokont algae)	Thiel, unpublished
Phaeovirus potassium channel KEsV	NP_077708.1	124 (3)	Ectocarpus siliculosus virus 1 (EsV-1)	Phycodnaviridae (Genus Phaeovirus)	355.593 (AF204951.1)	*Ectocarpus siliculosus** (Brown algae)	Balss et al., 2008
Poxvirus GAAP	NP_570396.1	237 (7)	Camelpox virus	Poxviridae (Chordopoxvirinae, Orthopoxviruses)	205.719.1 (NC_003391.1)	*Camelus* sp. (Mammal)	Saraiva et al., 2013
Pbi chlorovirus aquaglyceroporin	ABT13584.1	270 (6)	Chlorella Pbi virus MT325	see above	see above	see above	Gazzarrini et al., 2006
Pbi chlorovirus potassium transporter HAKCV	ABT15395.1	660 (12)	Chlorella Pbi virus Fr483	unassigned, Phycodnaviridae (Genus chlorellavirus)	321.240 (NC_008603.1)	see above	Greiner et al., 2012
SAG chlorovirus potassium transporter HAKCV	YP_001427177.1	644 (12)	Acanthocystis turfacea chlorella virus-1 (ATCV-1)	see above	see above	see above	Greiner et al., 2012
Ostreococcus tauri ammonium transporter	AFC35023.1	439 (11)	Ostreococcus tauri virus RT-2011 (OtV6)	Phycodnaviridae	189.567 (JN225873.1)	see above	Monier et al., 2017
Chlorovirus Ca-ATPase	ABT14089.1	871 (10)	Chlorella Pbi virus MT325	see above	see above	see above	Bonza et al. 2010

Table S2. Viral membrane transport proteins found in the databases. Alga species are indicated by *.

Putative protein	Example protein NCBI Accession #	Virus (virus family)	Length in aa, (Predicted TMs)	Genome size in base pairs, (accession #)	Host (Phylum)
Potassium channel	YP_009325597.1	Chlorovirus OSy-NE5 (phycodnaviridae, chlorovirus)	94 (2)	327.147 (NC_032001)	*Chlorella variabilis* Syngen 2-3* (Green algae)
Potassium channel	AUT19143.1	Dishui lake phycodnavirus 1 (phycodnaviridae)	116 (2)	181.035 (NC_037057.1)	Unknown alga*
Potassium channel	YP_009174599.1	Yellowstone lake phycodnavirus (phycodnaviridae)	96 (2)	171.045 (NC_028110.1)	Unknown alga*
Potassium channel	AUF82121.1	Tetraselmis virus 1 (TeV-1) (Mimiviridae)	81 (2)	668.031 (KY322437.1)	*Tetraselmis* sp.* (Green alga)
Potassium channel	ACH62227.1	Mycobacterium phage Myrna (Caudovirales, myoviridae)	119 (2)	164.602 (NC_011273.1)	Mycobacterium (Actinobacteria)
Potassium channel	AFN37377.1	Vibrio phage phi-pp2 (Caudovirales, Myoviridae)	228 (2)	246.421 (JN849462)	*Vibrio parahaemolyticus* (Proteobacteria)
Potassium channel	AND75308.1	Acinetobacter phage vB_AbaM_ME3 (unclassified phage)	124 (2)	234.900 (KK935715)	*Acinetobacter baumannii* DSM 30007 (Proteobacteria)
Potassium channel	YP_009282368.1	Lactobacillus phage PLE2 (Caudovirales, Siphoviridae)	259 (4)	35.068 (NC_031036)	*Lactobacillus casei* BL23 (Firmicutes)
CLIC-like chloride channel	YP_003358251	Anguillid herpes virus 1 (Herpesvirales (Alloherpesvirales, Cyprinivirus)	281 (2)	248.526 (NC_013668.3)	*Anguilla anguilla (*Fish)
MCLC-like chloride channel	YP_006908742	Abalone herpesvirus Victoria/AUS/2009 (Herpesvirales, unclassified)	333 (3)	211.518 (NC_018874.1)	*Haliotis rubra* (Molluscs)
Glutamate receptor	NP_048510.1	Paramecium bursaria chlorella virus-1 (Phycodnaviridae, chlorellavirus)	411 (3)	330.661 (JF411744)	*Chlorella variabilis* NC64A* (Green algae)
Ligand-gated ion channel	NP_048511.1	Paramecium bursaria chlorella virus-1 (Phycodnaviridae, chlorellavirus)	433 ($)	see above	see above
Aquaglyceroporin	YP_009325532.1	Chlorovirus OSy-NE5	269	see above	see above

		(phycodnaviridae, chlorovirus)	(6)		
Mechanosensitive channel	YP_009052121.1	Aureococcus anophagefferens virus isolate BtV-01 (Phycodnaviridae)	261 (3)	370.920 (NC_024697)	*Aureococcus anophagefferens** (Heterokont algae)
Mechanosensitive channel	AUF82136.1	Tetraselmis virus 1 (TeV-1) (Mimiviridae)	101 (1-2)	668.031 (KY322437.1)	*Tetraselmis* sp.* (Green algae)
Mechanosensitive channel	YP_003969926.1	Cafeteria roenbergensis virus BV-PW-1 (Mimiviridae)	101 (2)	617.453 (NC_014637.1)	*Cafeteria roenbergensis* strain E4-10* (Heterokont algae)
Ammonium transporter	YP_009173407.1	Chrysochromulina ericina virus (Phycodnaviridae)	124 (2)	473.558 (NC_028094.1)	*Chrysochromulina ericina** (Haptophyte algae)
Magnesium transporter	YP_009222254.1	Lactobacillus phage Lfelnf (Caudovirales, myoviridae)	322 (2/3)	106.071 (NC_029058.1)	*Lactobacillus fermentum* (Firmicutes)
Sodium calcium transporter	AUR86274.1	Vibrio phage 1.084.O. 10N.261.49.F5 (unclassified)	370 (9)	141.906 (MG592459.1; partial genome)	*Enterovibrio norvegicus* (Proteobacteria)
Sodium phosphate symporter	AEO97646.1	Emiliania huxleyi virus 84 (Phycodnaviridae, coccolithoviruses)	534 (10)	396.620 (JF974290.1)	*Emiliania huxleyi* 1516* (Heterokont algae)
Sodium glucose transporter	NP_932589.1	Aeromonas virus 44RR (Caudovirales, myoviridae)	507 (14)	173.591 (NC_005135.1)	*Aeromonas salmonicida* (Protobacteria)
short ATP-binding casette transporter (ABC transporters)	BAV62853.1	Acanthamoeba castellanii mimivirus shirakomae (Mimiviridae)	532 (5)	1.182.849 (AP017645.1 (check))	*Acanthamoeba castellanii* (Amoebozoa)
large ATP-binding casette transporter (ABC transporters)	YP_009001498.1	Anomala cuprea entomopoxvirus (Poxviridae entomopoxvirinae, Alphaentomopoxvirus)	1506 (14)	245.717 (NC_023426.1)	*Anomala cuprea* (Insect)
Nicotinamide mononucleotide transporter	AKJ73485.1	Salmonella phage 40 (unclassified bacterial virus)	239 (6)	63.263 (KR296694.1)	*Salmonella paratyphi* A (Proteobacteria)
Equilibrative nucleoside transporter	AJG42984.1	Harp seal herpesvirus (Herpesvirales, herpesviridae, gammaherpesviridae)	432 (11)	117.276 (partial) (KP136799.1)	*Pagophilus groenlandicus* (Mammal)
Amino acid transporter	BAZ95678.1	Red seabream iridovirus (Iridoviridae Alphairidovirinae; Megalocytivirus))	378 (11)	112.590 (AP017456.1)	*Pagrus major* (Fish)
Ca-ATPase	YP_009325865.1	Chlorovirus OSy-NE5	$	see above	*see above*

		(Phycodnaviridae, chlorovirus)			
Mg-ATPase	ARF11485.1	Klosneuvirus-1 (Mimiviridae, Klosneuvirinae)	922 (10)	451.653 (KY684108.1)	Unknown/Environmental sample
P-type ATPase	ARF08724.1	Catovirus CTV1 (Mimiviridae, Klosneuvirinae)	809 (6-7)	1.152.313 (KY684083.1)	Unknown/Environmental sample

Table S3. Putative potassium channel proteins from vibrio phages.

Phage	Length (aa)	TMs	Accession #
Vibrio phage phi-pp2	228	2	AFN37377.1
Vibrio phage nt-1	228	2	YP_008125342.1
Vibrio phage ValKK3	227	2	YP_009201217.1
Vibrio phage VH7D	207	2	YP_009006109.1
Vibrio phage phi-Grn1	200	2	ALP47069.1
Vibrio phage phi-ST2	161	1	ALP47495.1
Vibrio phage 1.081.O._10N.286.52.C2	219	2	AUR85893.1
Vibrio phage 1.084.O._10N.261.49.F5	219	2	AUR86273.1
Vibrio phage 2.275.O._10N.286.54.E11	149	2	AUS02797.1

Table S4. Putative potassium channel-like proteins from large DNA viruses.

Virus	Length (aa)	TMs	Accession #
Bandra mega virus	114	2	AUV58190.1
Megavirus courdo11	114	2	AFX92280.1
Megavirus courdo7	114	2	AEX61336.1
Powei lake megavirus	114	2	ANB50367.1
Moumouvirus Monve	113	2	AEX63115.1
Moumouvirus australiensis	113	2	AVL94583.1
Acanthamoeba polyphaga mimivirus	111	2	YP_003987221.1
	111	2	AKI80434.1
Hirudovirus strain Sangsue	127	2	AHA45141.1
Tupanvirus deep ocean	103	2	AUL80003.1

Table S5. Blastp search results of the MCLC-like protein from Abalone herpesvirus against sequences from cellular organisms; *partial sequence.

Organism (Phylum)	length (aa)	max score/total score/query cover (%)/E-value/identity (%)/ accession #
Lingula anatina (Brachiopoda) isoform X2	441	92.8/92.8/47/2e-17/32/XP_13403059.1
Lingula anatina	467	93.2/93.2/47/2e-17/32/XP_013416639.1
Lingula anatina isoform X1	467	92.8/92.8/47/2e-17/32/XP_013403058.1
Branchiostoma floridae (Chordata)	685	87.0/87.0/51/3e-15/26/XP_002610653.1
Saccoglossus kowalevskii (Hemichordata)	196*	78.2/78.2/45/2e-13/33/XP_006819247.1
Branchiostoma belcheri (Chordata)	573	72.4/72.4/50/2e-10/24/XP_019625644.1
Acropora digitifera (Cnidaria)	441	68.9/68.9/81/2e-9/22/XP_015775446.1
Orbicella faveolata (Cnidaria)	523	66.6/66.6/50/2e-8/25/XP_020611811.1
Hydra vulgaris (Cnidaria)	576	60.8/60.8/47/1e-6/26/XP_002154667.2
Lingua anatina (Brachiopoda)	475	60.1/60.1/49/2e-6/28/XP_013385450.1
Lingua anatina	366	59.3/59.3/49/3e-6/28/XP_013408110.1
Intoshia linei (Orthonectida)	389	56.6/56.6/30/2e-5/24/OAF71881.1
Crassostrea gigas (Mollusca)	265	55.1/55.1/35/5e-5/23/EKC19861.1
Crassostrea gigas	354	55.5/55.5/39/5e-5/24/ XP_011419399.1
Lottia gigantea (Mollusca)	616	53.9/53.9/39/2e-4/22/XP_009044054.1
Strongylocentrotus purpuratus (Echinodermata)	347*	53.1/53.1/37/3e-4/28/XP_011676168.1
Diachasma alloeum (Arthropoda)	501	52.4/52.4/68/6e-4/23/XP_015115510.1

Table S6. Results of blastp search of camel pox virus GAAP against viral protein sequences.

Virus	length (aa)	TMs	E value	aa identity	Accession #
Camelpox virus	237	7	-	-	NP_570396.1
	237	7	2e-175	99%	AAG37461.1
Cowpox virus	237	7	1e-169	97%	ADZ29755.1
	237	7	7e-169	96%	SNB58186.1
	237	7	1e-168	96%	SNB53700.1
	237	7	3e-168	96%	ADZ30397.1
	237	7	1e-160	96%	ADZ30183.1
	237	7	2e-160	96%	CRL86727.1
	237	7	2e-158	95%	AQQ13071.1
	237	7	5e-158	95%	ADZ24213.1
	210	6	1e-142	95%	CAD90752.1
	199	5	5e-129	95%	CRL87022.1
	197	5	2e-127	95%	SNB49536.1
	114	3	1e-77	96%	ABD97561.1
	79	2	2e-41	94%	ABD97560.1
Vaccinia virus	237	7	1e-158	95%	AAV98625.1
	237	7	6e-158	95%	Q49P94.1
	237	7	4e-157	94%	AAW21699.1
Scale drop disease virus	236	7	1e-80	47%	YP_009163856.1
Monkeypox virus	121	4	6e-64	80%	AAY96807.1
	105	3	3e-55	80%	AKG51345.1
	105	3	1e-54	81%	AGR36454.1
	105	3	6e-54	80%	NP_536611.1
	105	3	9e-54	80%	AAY97191.1
Harp seal herpesvirus	244	6	4e-61	48%	AJG42933.1
uncultured Caudovirales phage)	233	7	5e-15	30%	ASN71498.1
Panine betaherpesvirus 2	254	7	1e-10	27%	NP_612793.1

T**able S7.** Blastp results of camel poxvirus GAAP versus living organisms.

Hit	Max score/Total score/Query cover/E value/Ident/Accession
protein lifeguard 4 (*Castor canadensis*)	353/353/100%/2e-121/75%/XP_020024307.1
protein lifeguard 4 isoform X1 (*Odocoileus virginianus texanus*)	350/350/97%/7e-120/76%/XP_020738958.1
protein lifeguard 4 isoform X1 (*Condylura cristata*)	349/349/100%/2e-119/74%/XP_012576116.1
protein lifeguard 4 (*Ovis aries musimon*)	348/348/97%/4e-119/76%/XP_011995634.1
protein lifeguard 4 (*Capra hircus*)	347/347/97%/7e-119/76%/XP_017903468.1
protein lifeguard 4 (*Bos taurus*)	347/347/97%/1e-118/76%/NP_001014914.1
protein lifeguard 4 (*Physeter catodon*)	346/346/100%/2e-118/74%/XP_007126330.1
protein lifeguard 4 isoform X1 (*Desmodus rotundus*)	346/346/97%/2e-118/74%/XP_024431972.1
protein lifeguard 4 (*Ovis aries*)	345/345/96%/4e-118/76%/XP_014950252.1
protein lifeguard 4 (*Bison bison bison*)	345/345/97%/4e-118/76%/XP_010839876.1

Table S8. ABC transporter diversity among large DNA viruses. Shown are the results of a blastp search of the mimivirus ABC transporter (YP_003987262.1) against virus sequences.

Virus (genome size; accession #)	length (aa)	TMs	E value	aa identity	Accession #
Acanthamoeba polyphaga mimivirus	532	5	0.0	100%	YP_003987262.1
(1.181.404; AY653733.1)	532	5	0.0	99%	AKI79520.1
	532	5	0.0	98%	AKI81410.1
	532	5	0.0	93%	AKI80473.1
	532	5	0.0	98%	AEJ34979.1
	532	5	4e-122	100%	AEJ34978.1
	532	5	4e-87	36%	AVG45935.1
	532	5	3e-86	35%	AVG47038.1
Acanthamoeba castellanii mamavirus (1.191.693 bp; JF801956.1)	532	5	0.0	99%	AEQ60944.1
Acanthamoeba polyphaga moumouvirus (1.021.348 bp; NC_020104.1)	535	5	1e-91	37%	YP_007354121.1
Moumouvirus Monve (no full genome sequence)	492	1-4	1e-90	36%	AEX63154.1
Megavirus courdo11 (1.246.126 bp; JX975216.1)	542	4	2e-88	35%	AFX92233.1
Powei lake megavirus (1.208.707 bp; KU877344.1)	526	3-4	7e-88	35%	ANB50323.1
Megavirus courdo7 (no full genome sequence)	526	3-4	3e-87	35%	AEX61283.1
Megavirus Iba (1.230.522 bp; JX885207.1)	599	3-4	3e-86	35%	AGD92104.1
Megavirus chiliensis (1.259.197 bp; NC_016072.1)	605	3	4e-86	35%	YP_004894252.1
Megavirus vitis (1.242.360 bp; MG807319.1)	484	1-2	2e-84	36%	AVL93548.1

Bandra megavirus (73.092 bp (partial); MG779341.1)	535	3-4	3e-82	36%	AUV58153.1
Moumouvirus australiensis (1.098.002 bp; MG807320.1)	534	5	7e-82	36%	AVL94552.1
Moumouvirus goulette (1.016.844 bp; KC008572.1)	535	5	9e-76	34%	AGF85616.1
Tupanvirus deep ocean	572	4-5	5e-16	32%	AUL79468.1
(1.516.267 bp; MF405918.1)	545	3	2.9	31%	AUL79194.1
Catovirus CTV1 (1.152.313 bp; KY684083.1)	545	5	4e-7	24%	ARF09084.1
Indivirus ILV1 (267.262; KY684085.1)	547	5	0.008	31%	ARF09888.1
Hokovirus HKV1 (450.695 bp; KY684103.1)	551	5	0.012	23%	ARF10734.1
Bodo saltans virus (1.385.869 bp; MF782455.1)	452	4	0.029	24%	ATZ80684.1
Tupanvirus soda lake (1.439.508 bp; KY523204.1)	545	3	2.4	29%	AUL77901.1

Table S9. Putative sodium phosphate transporters from viruses that infect marine picoplankton; *possibly partial sequence.

Virus	Length (aa)	TMs	Accession #
Emiliania huxleyi virus 86	534	10	YP_293871.1
Emiliania huxleyi virus 203	534	10	AEO98102.1
Emiliania huxleyi virus 86	508*	9	YP_002296186.1
Emiliania huxleyi virus 202	534	10	AET42514.1
Bathycoccus sp. RCC1105 virus BpV1	503	9	YP_004061633.1
Ostreococcus lucimarinus virus 2	459	9	YP_009172747.1
Ostreococcus tauri virus 2	459	9	YP_004063655.1
Ostreococcus lucimarinus virus OlV4	290*	5	AET84692.1
Ostreococcus lucimarinus virus 1	459	9	YP_004061866.1
Ostreococcus lucimarinus virus OlV4	176*	3-5	AET84699.1

Table S10. Phage nicotinamide mononucleotide transporter variety. Blastp comparison of Salmonella phage 40 protein against viral sequences.

Phage	Length (aa)	TMs	blastp E value (compared to the protein from Salmonella phage 40)	aa identity	Accession #
Salmonella phage 40	239	6-7		100%	AKJ73485.1
Salmonella phage PVO-SE1	242	6-7	1e-157	94%	YP_004893838.1
Klebsiella phage vB KpnM KB57	240	7	4e-154	89%	YP_009187643.1
Klebsiella phage vB KpnM BlS47	240	7	6e-154	89%	ARB12513.1
Salmonella phage SSE121	237	8	7e-152	89%	YP_009148852.1
Raultella phage Ro1	233	6-7	1e-138	83%	AUE23444.1
Cronobacter phage vB CsaM GAP31	235	6-7	4e-137	82%	YP_006987079.1
Escherichia phage 4MG	240	6-7	1e-131	77%	YP_008857242.1
Yersinia phage fHe-Yen9-03	267	8	6e-89	60%	SOK59171.1
Yersinia phage fHe-Yen9-04	267	8	4e-88	60%	SOK58638.1
Serratia phage BF	267	7-8	2e-86	60%	AQW88885.1
Pectobacterium phage CBB	267	7	7e-86	61%	AMM43925.1
Cronobacter phage vB CsaM GAP 32	267	7	2e-85	60%	YP_006987459.1
Bacillus phage PBC2	228	8	5e-44	38%	AKQ08367.1
Salmonella phage 100268_sal2	225	7	2e-40	39%	YP_009320809.1
Escherichia virus EPS7	225	7	3e-40	39%	YP_001837008.1
Salmonella virus Stitch	225	7	3e-40	39%	YP_009146019.1
Salmonella phage BSP22A	225	7	1e-39	39%	ARM69751.1
Bacteriophage T5-like chee130_1	225	7	1e-39	39%	ASU02438.1
Bacillus phage vB BanS-Tsamsa	228	8	2e-38	35%	YP_008873313.1
Yersinia phage phiR201	225	7	2e-38	38%	YP_007237046.1
Vibrio phage 11895-B1	231	8	8e-38	36%	YP_007673552.1
Vibrio phage S4-7	234	7	1e-36	39%	AOQ26732.1
Lactobacillus phage Bacchae	256	6-7	2e-17	31%	AUV59971.1
Lactobacillus phage Semele	256	6-8	8e-17	32%	AUV60242.1
Lactobacillus phage LpeD	256	8	1e-16	30%	ATG86388.1
Lactobacillus virus LP65	256	6-8	2e-15	30%	YP_164718.1

Cronobacter phage vB CsaP GAP52	207	7-8	6e-13	27%	YP_006987679.1
Vibrio virus Thalassa	208	8	8e-13	29%	AUG85311.1
Vibrio phage 2.275.O._10N.286.54.E11	246	7-8	2e-11	24%	AUS02861.1
Vibrio virus Ceto	208	7-8	3e-11	26%	AUG85113.1
Vibrio phage pVp-1	210	6	5e-11	27%	YP_007007886.1
Enterococcus phage EFDG1	263	7-8	3e-5	23%	YP_009218286.1
Enterococcus phage EFP01	254	7-8	3e-5	24%	APZ82054.1
Enterococcus phage EF1	256	8	2e-4	24%	ASZ76735.1

Table S11. Phage channels and transporters that are identical to a protein from a bacterium.

Phage	Protein type	Accession # (phage protein)	Organism	Accession # (bacterial protein)
Lactobacillus phage PLE2	Potassium channel	YP_009282368.1	*Lactobacillus casei*	WP_012491300.1
Streptococcus phage phiZJ20091101-4	CLC-like chloride channel	ANM47568.1	*Streptococcus suis*	WP_024390438.1
Streptococcus phage phi-SsuD.1	Cu-translocating P-type ATPase	CBR26873.1	*Streptococcus sp.*	WP_000086363.1
Dickeya phage phiDP10.3	Malate/Na symporter	AIM51479.1	*Dickeya solani*	WP_02263587.1
Burkholderia virus phi1026b	MFS transporter	AAR23210.1	*Burkholderia pseudomallei*	WP_004552955.1
Flavobacterium phage 6H	Cytochrome c oxidase	YP_008320461.1	*Flavobacterium psychrophilum*	WP_034099077.1
Staphylococcus phage UPMK_1	Arsenical pump	ATW68282.1	*Staphylococcus aureus*	WP_000989117.1
Leptospira phage vB_LinZ_10-LE1	RND transporter, Heavy Metal Efflux (HME) family	AGS80748.1	*Leptospira inadai*	WP_010420104.1

Figure S1

A

```
YSLV          -------------------------------------MFHNFFKLLVALIVT--------   15
OLPV2         -------------------------------------MKNKFIYLIVSTIFFSLVYSFIR   23
AaV1          -----------------------MKLLFGYN---------RFHLLIYQIIFFSILYMFLG   28
TeV1          -----------------------------------MSFVAKVAVHIILIITFFVINLTIN   25
PBCV1         --------------------------MLVFSKFL--TRTEPFMIHLFILAMFVMIYKFFP   32
OSyNE5        --------------------------MFIIRKIL--TQSEPFIIHLLVLMMFVMIYRFFP   32
MT325         --------------------------------------MSILGVHFAILLLFAALYKFFP   22
ATCV1         --------------------------------------MLLLIIHIIILIVFTAIYKMLP   22
OtV6          -----------------------------------MALTKTLSLNFISILLFTLMYFTIS   25
DishuiLV      ---------------------------------MASTLARTVGLNLGAIFIFAILYLALA   27
EsV1          MSRRLFATCGIAIALRGLVVSGGVKEIVSFRPLIDTSLVGGILSNLILLVVFAELYWQLD   60
BpV1          ------------------------------------MLNKAVTIALIITLVYGYLY----   20
MpV1          --------------------------------------MKTVLNILIITTLYGLLY----   18
                                                       .   :
YSLV          --------------------------------NLSFAGVMHSWVTDSDM-----------   32
OLPV2         PRNFHGFNKIQDKIQDVLVSEETDIIEPFYAP----------YLDKKE--KEKEKEKEKD   71
AaV1          SSHFSGINTLEDILKNEIVSKQV--LDPIIEEKFTNASDPSKFISKDDIEVDKKETEEIQ   86
TeV1          ITNK---------------------------NAFS-------------------------   33
PBCV1         GGFE---------------------------NNFSVA-----------------------   42
OSyNE5        GGFE---------------------------NNFTVA-----------------------   42
MT325         GGFE---------------------------NNFKRG-----------------------   32
ATCV1         GGMF---------------------------SN---------------------------   28
OtV6          KAGG---------------------------EQFNGL-----------------------   35
DishuiLV      RMGT---------------------------EDFVGM-----------------------   37
EsV1          QGDD---------------------------HTHFGF-----------------------   70
BpV1          STMK---------------------------EDFGFS-----------------------   30
MpV1          SKMK---------------------------PEHFNF-----------------------   28

YSLV          ----------------SGLRKGSTDRFVDLLYFSIVSFSTTGYGDIAPKSTRAKMAVCLF   76
OLPV2         EVKKEVKENVLEEKEKI-YNPSWWQHYLDSLYFSVITSCLLGYGDIYPITNMSKIIVSLQ   130
AaV1          EKAKEIKKEVKKELNILTDKDSFFDRFFLRFYFSFVTSTTIGYGDTTPSSISTRTLAMIQ   146
TeV1          ----------------------KELTTLDVFYYTVTTWTTTGYGDIYPVITVSKMIAVTK   71
PBCV1         ------------------NPD-KKASWIDCIYFGVTTHSTVGFGDILPKTTGAKLCTIAH   83
```

```
OSyNE5          ------------------NPD-KKASFVDCLYFGVTTHSTVGFGDILPKTTGAKLCTIAH  83
MT325           ------------------DGSKEPVSWMDAIYVSAATHTTTGFGDIVADSRAAKFAVTAH  74
ATCV1           ----------------------TDPTWVDCLYFSASTHTTVGYGDLTPKSPVAKLTATAH  66
OtV6            ---------------------DKDSGFLDHLYFAFTVQSTVGFGDIYPISPMAKMIVMVQ  74
DishuiLV        ---------------------DRMSSPLDALYLSMTVQSTIGFGDITPKTTRAKLLVMMQ  76
EsV1            ------------------------SSAIDAYYFSAVTSSSVGYGDLLPKTPKAKLLTIAH  106
BpV1            ------------------------DDPLDPYYFSLMTMSTVGYGDFSPKTRRAKALVMSH  66
MpV1            ------------------------KSPLDPFYFSFTTMSSVGYGDFSPKTDAAKLVVMSQ  64
                                           .   *         *:**       ::  .

YSLV            LMFV--NI---AAIYGIYNALVTSA---------------  96
OLPV2           GFITLFLILS------------------------------  140
AaV1            ACSTFYILMA------------------------------  156
TeV1            MLLFLVILMY------------------------------  81
PBCV1           IVTVFFIVLTL-----------------------------  94
OSyNE5          IVVVFFIVLTL-----------------------------  94
MT325           MLIVFSIVVLGLKPELITNLI-------------------  95
ATCV1           MLIVFAIVISGFTFPW------------------------  82
OtV6            QSVLILGILELISESKSVANVVPTVMKKMI----------  104
DishuiLV        QFVVIVGIVNLLSGGGISLKKNNAAMNTISNTISNVPAPA  116
EsV1            ILAMFFVMLPVVAKALEK----------------------  124
BpV1            HTVILVELATILSKMTK-----------------------  83
MpV1            HLVMIGELAKILKIF-------------------------  79
```

B

```
Vp2             ------------------------------------------------------------    0
Vp3             ------------------------------------------------------------    0
Moraxella       ------------------------------------------------------------    0
Vp1             ------------------------------------------------------------    0
VpVH7D          ------------------------------------------------------------    0
VpGrn1          ------------------------------------------------------------    0
VpValKK3        ------------------------------------------------------------    0
VpST2           ------------------------------------------------------------    0
Vpphipp2        ------------------------------------------------------------    0
Vpnt1           ------------------------------------------------------------    0
```

```
LpPLE2            MATKKVNKVLIALHKYYTVLFAILALLSVALIVLDYMGRISIDKSPYTEIDNGILVIFAI   60
Lactobacillus     MATKKVNKVLIALHKYYTVLFAILALLSVALIVLDYMGRISIDKSPYTEIDNGILVIFAI   60
MpMyrna           ------------------------------------------------------------   0
MpPhabba          ------------------------------------------------------------   0
SpBillNye         ------------------------------------------------------------   0
Acidobacterium    ------------------------------------------------------------   0
Ptexasensis       ------------------------------------------------------------   0
Rheinheimera      ------------------------------------------------------------   0
Ap                ------------------------------------------------------------   0
Ppventosus        ------------------------------------------------------------   0
YSLV              ------------------------------------------------------------   0
OLPV2             ------------------------------------------------------------   0
AaV1              -----------------------------------------------------MKLLFGY   7
TeV1              ------------------------------------------------------------   0
PBCV1             --------------------------------------------------------MLVF   4
OSyNE5            --------------------------------------------------------MFII   4
MT325             ------------------------------------------------------------   0
ATCV1             ------------------------------------------------------------   0
OtV6              ------------------------------------------------------------   0
DishuiLV          ------------------------------------------------------------   0
EsV1              ---------------MSRRLFATC---G---IAIALRG---------LVVSGGVKEIVSF   30
BpV1              ------------------------------------------------------------   0
MpV1              ------------------------------------------------------------   0


Vp2               ------------------------------------------------------------   0
Vp3               ------------------------------------------------------------   0
Moraxella         ------------------------------------------------------------   0
Vp1               ------------------------------------------------------------   0
VpVH7D            ------------------------------------------------------------   0
VpGrn1            ------------------------------------------------------------   0
VpValKK3          ------------------------------------------------------------   0
VpST2             ------------------------------------------------------------   0
Vpphipp2          ------------------------------------------------------------   0
Vpnt1             ------------------------------------------------------------   0
LpPLE2            DYFSRMLHADSKWDFFKHNLIDLLAIIPFNYAFSFFRFGRIFRLARLTRLMRLTRLARLA   120
```

```
Lactobacillus       DYFSRMLHADSKWDFFKHNLIDLLAIIPFNYAFSFFRFGRIFRLARLTRLMRLTRLARLA   120
MpMyrna             ------------------------------------------------------------   0
MpPhabba            ------------------------------------------------------------   0
SpBillNye           ------------------------------------------------------------   0
Acidobacterium      ------------------------------------------------------------   0
Ptexasensis         ------------------------------------------------------------   0
Rheinheimera        ------------------------------------------------------------   0
Ap                  ------------------------------------------------------------   0
Ppventosus          ------------------------------------------------------------   0
YSLV                --------------MFHNFFKLLVALIVT-------------------------------   15
OLPV2               --------------MKNKFIYLIVSTIFFSLVYSFIRPRNFHGFNKIQDKIQDVLVSEET   46
AaV1                N----------------RFHLLIYQIIFFSILYMFLGSSHFSGINTLEDILKNEIVSKQV   51
TeV1                -----M---S----FVAKVAVHIILIITFFVINLTINITNK-------------------   29
PBCV1               SKFL-----T----RTEPFMIHLFILAMFVMIYKFFPGGFE-------------------   36
OSyNE5              RKIL-----T----QSEPFIIHLLVLMMFVMIYRFFPGGFE-------------------   36
MT325               ---------------MSILGVHFAILLLFAALYKFFPGGFE-------------------   26
ATCV1               ---------------MLLLIIHIIILIVFTAIYKMLPGGMF-------------------   26
OtV6                -----M---A----LTKTLSLNFISILLFTLMYFTISKAGG-------------------   29
DishuiLV            ---MAS---T----LARTVGLNLGAIFIFAILYLALARMGT-------------------   31
EsV1                RPLIDT---S----LVGGILSNLILLVVFAELYWQLDQGDD-------------------   64
BpV1                ---------M----LNKAVTIALIITLVYGYLY----STMK-------------------   24
MpV1                ---------------MKTVLNILIITTLYGLLY----SKMK-------------------   22


Vp2                 ------------------------MLNCITVID----SHGRKEYKTSFLLLVN-IVTVLL   31
Vp3                 ----------------MKKYLHWCISTSSKMYK----KYGT-----KYILILT-GSITGL   34
Moraxella           ----------------MSRF---TLSKRIYRTD----EFEVRNYNMLFLTFVAVVILYLS   37
Vp1                 --------------------------MKFKSYD----SFGVVEYNIGMIFAGL-MALSFT   29
VpVH7D              ---------------------------------------------MLMIFGLM-IVIALS   14
VpGrn1              ----------------------------------------------------M-IVIALS   7
VpValKK3            ----------------MKRF-----KKHIRKYD----EHGVAQYNMLMIFGLM-IVIALS   34
VpST2               ------------------------------------------------------------   0
Vpphipp2            ----------------MKRF-----KKHIKKYD----EHGVVQYNMLMIFALI-VTISMS   34
Vpnt1               ----------------MKRL-----KKHIRKYD----EHGVVQYNMLLIFALI-ISISTS   34
LpPLE2              GIVGI-----LTK---HAE------------------RILKRTGLIYYI------WLSAA   148
Lactobacillus       GIVGI-----LTK---HAE------------------RILKRTGLIYYI------WLSAA   148
```

```
MpMyrna          -----------------MS------------------LIHRATGSIKVV-----VGWAIG   20
MpPhabba         -----------------MS------------------KIHDATGSVAKV-----GLWAVG   20
SpBillNye        ----------------MKR------------------VFFRLANTTHML-----VLTIIG   21
Acidobacterium   ----------MSN---INK------------------LIARATDSFPEI-----IAYYLA   24
Ptexasensis      ----------------MKK------------------LLIYIANRLWLI-----LAIYAG   21
Rheinheimera     ----------------MKK------------------LLIYIANRLWLI-----IALYAA   21
Ap               -----------------MA------------------YFLLIANNIRYV-----FLAYLL   20
Ppventosus       ----------------MKR------------------SILWLSNSIWRV-----FGLYLI   21
YSLV             ---------NLSFAGVMHSWVT---DSDM-------------------------------   32
OLPV2            DIIEPFYAP----------YLD---KKE--KEKEKEKEKDEVKKEVKEN-----VLEEKE   86
AaV1             --LDPIIEEKFTNASDPSKFIS---KDDIEVDKKETEEIQEKAKEIKKE-----VKKELN   101
TeV1             --------NAFS------------------------------------------------   33
PBCV1            --------NNFSVA----------------------------------------------   42
OSyNE5           --------NNFTVA----------------------------------------------   42
MT325            --------NNFKRG----------------------------------------------   32
ATCV1            --------SN--------------------------------------------------   28
OtV6             --------EQFNGL----------------------------------------------   35
DishuiLV         --------EDFVGM----------------------------------------------   37
EsV1             --------HTHFGF----------------------------------------------   70
BpV1             --------EDFGFS----------------------------------------------   30
MpV1             --------PEHFNF----------------------------------------------   28


Vp2              NIYIGGLFLWHFEEGLQDSNINSLKEAMWAVFMTMTTIGFGDKYPITVEGYITTGVCFLL   91
Vp3              NVIIGALFLYLFEHNVDNALINSYSSALWMNWMVATTVGFGDYYPISTGGKIVTGISSAI   94
Moraxella        IAV-LAFLLLWAESGAQGANIETYGDAFWTLQMSASTIGFGDYYPVTLGGRTIVAAMFYI   96
Vp1              IALTAASLLYRFESGADGSNINSAGDALWTIWMSMSTIGFGDHYPVTVGGRVIVGSMFAV   89
VpVH7D           IALTAANILFSFEIQNPDANITTPGDSFWTVWMAMSTIGFGDKYPVTTGGRYVIGCMFVV   74
VpGrn1           IALTAANILFSFEIQNPDANITTPGDSFWTVWMAMSTIGFGDKYPVTTGGRYVIGCMFVV   67
VpValKK3         IALTAANILFSFEIQNPDANIKTPGDSFWTVWMAMSTIGFGDKYPVTTGGRYVIGCMFVV   94
VpST2            --------------------------------MAMSTIGFGDKYPVTTGGRYVIGCMFVV   28
Vpphipp2         IALVASNILFAFELSASNANIKTPGDAFWTVWMAMSTIGFGDHYPVTTGGRFVIGSMFVV   94
Vpnt1            IAFIAANFLFYFEVQDPNANIRTYGDAFWTVWMAMSTIGFGDHYPVTEGGRYTIGCMFVV   94
LpPLE2           LILIGASVYSVT------ESVG-YGDSLWWAIVTATTVGYGDISPHTVLGRIAAVILMFN   201
Lactobacillus    LILIGASVYSVT------ESVG-YGDSLWWAIVTATTVGYGDISPHTVLGRIAAVILMFN   201
MpMyrna          CTIAGAVAFCLF------EGSTDGLAALYWAVVSGSSTGYGDIAPKTAAGRITTIVYLIV   74
```

```
MpPhabba            FTVAGTLAYSLF------EHVT-PFESLYWAIVSGSSTGYGDYSPATVGGRVVTIVYLVI   73
SpBillNye           TILVSSVVFSLT------EGVS-LFDGVYWSVVTMSTTGYGDLSPESIVGKAYTMLLMLW   74
Acidobacterium      TLTVSGVLFAYF------EGKP-LFESFWWACVTGLTIGYGDMYPVTVGGKIVAIVLMHV   77
Ptexasensis         SLIFGAISFAVI------EQKS-LADGIWWATVTSLTIGYGDLTPVTPIGRFVGILFGHF   74
Rheinheimera        SLIFGAVSFAIL------EQKS-LADGLWWATVTSLTIGYGDLTPVTPAGRIVGILFGHF   74
Ap                  SLVIASLLFSLL------EGVS-YLDSLYWACVTSLTIGYGDYSPHTIAGKILAIVCGHF   73
Ppventosus          SVVLCALLFAWI------ESKS-VLDSIWWACVTSLTIGYGDIAPVTTAGRALAVVFSHF   74
YSLV                ------SGLRK-------GSTDRFVDLLYFSIVSFSTTGYGDIAPKSTRAKMAVCLFLMF   79
OLPV2               K-----I-YNP-------SWWQHYLDSLYFSVITSCLLGYGDIYPITNMSKIIVSLQGFI   133
AaV1                I-----LTDKD-------SFFDRFFLRFYFSFVTSTTIGYGDTTPSSISTRTLAMIQACS   149
TeV1                -------------------KELTTLDVFYYTVTTWTTTGYGDIYPVITVSKMIAVTKMLL   74
PBCV1               --------NPD--------KKASWIDCIYFGVTTHSTVGFGDILPKTTGAKLCTIAHIVT   86
OSyNE5              --------NPD--------KKASFVDCLYFGVTTHSTVGFGDILPKTTGAKLCTIAHIVV   86
MT325               --------DGS-------KEPVSWMDAIYVSAATHTTTGFGDIVADSRAAKFAVTAHMLI   77
ATCV1               -------------------TDPTWVDCLYFSASTHTTVGYGDLTPKSPVAKLTATAHMLI   69
OtV6                ------------------DKDSGFLDHLYFAFTVQSTVGFGDIYPISPMAKMIVMVQQSV   77
DishuiLV            ------------------DRMSSPLDALYLSMTVQSTIGFGDITPKTTRAKLLVMMQQFV   79
EsV1                ----------------------SSAIDAYYFSAVTSSSVGYGDLLPKTPKAKLLTIAHILA   109
BpV1                ---------------------DDPLDPYYFSLMTMSTVGYGDFSPKTRRAKALVMSHHTV   69
MpV1                ---------------------KSPLDPFYFSFTTMSSVGYGDFSPKTDAAKLVVMSQHLV   67
                                                          *:**

Vp2                 GAVNLGTLIKTASNLVKD--KNEVDNRQIYSIITEL--LR--SNQHIEQELNLTQEV--T   143
Vp3                 GIGMFGLFTSVVSGILMEHTDDSIRNRELKEQNARI--EI--ELNEVKKIL-----VSFT   145
Moraxella           GVGIVGFIGAQVADRFLGFADTNVKNRELRKQNEQI--LE--HNKVLEKKLDKLLS----   148
Vp1                 GATLIGLNIGIASAWVTSKFDKSVQNRVLKLQSDTL--IQ--KIDRLETFLNVDQTVYFG   145
VpVH7D              GTSLLGLTIGIINSWVVSKFDKSIQNRVLKSQNDAL--LH--KLDRLETHLGIDQEVRFD   130
VpGrn1              GTSLLGLTIGIINSWVVSKFDKSIQNRVLKSQNDAL--LH--KLDRLETHLGIEQEVRFD   123
VpValKK3            GTSLLGLTIGIINSWVVSKFDKSIQNRVLKSQNDAL--LH--KLDRLETHLGIDQEVRFD   150
VpST2               GTSLLGLTIGIINSWVVSKFDKSIQNRVLKSQNDAL--LH--KLDRLETHLGIDQEVRFD   84
Vpphipp2            GASLIGLNIGIANAWITSKFDKSIQNRVLKSQNDAL--LQ--KLDRLETHLGIDQEVLFS   150
Vpnt1               GASLIGLNIGIVNGWISSKFDKSIQNRVLKSQNDAL--LQ--KLDRLETFLGVNQEVLFD   150
LpPLE2              GI---GLISALTSAVT----AYLSGTNSDSSQSPTDEIKKLYDLKQIGAITQSEYDAK--   252
Lactobacillus       GI---GLISALTSAVT----AYLSGTNSDSSQSPTDEIKKLYDLKQIGAITQSEYDAK--   252
MpMyrna             ML--WVITPVLTARIA----AYMIVNNDAWTDSEQESLKG--DIAAIKRKLGA-------   119
MpPhabba            ML--WVVTPVLTARIA----AYMIVNNDAWTDNEQETLKQ--DIADIKRKLGA-------   118
```

```
SpBillNye           SV--FFLVPSAVAQII----MKFIHNRNEFTHEEQEKIMQ--QQKRIIELLEGGKSE---   123
Acidobacterium      VP--LIIIPLIVARLL----STVIEDKNVFSDAEQEALKS--DIAAIKKALKIEKAKE--   127
Ptexasensis         WI--FVVIPMIVANII----MHLVEDKHLFSDEEQRELMQ--RLKRIEARLEQSKD----   122
Rheinheimera        WI--FIVIPMIVANII----MHLVEDKHLFSDEEQRELMQ--RLKRIESRLTQQEQDN--   124
Ap                  WI--FFIIPSVISHIL----AALIKNRNEFTHEEQEDIKE--SLSQIKCHLLKNQDDL--   123
Ppventosus          WI--FGVAPLVISNML----NITLEDRNLFTHEEQEEMKT--LLRKLAEKA---------   117
YSLV                V----NI---AAIYGI----YNALVTSA--------------------------------   96
OLPV2               TL--FLILS---------------------------------------------------   140
AaV1                TF--YILMA---------------------------------------------------   156
TeV1                FL--VILMY---------------------------------------------------   81
PBCV1               VF--FIVLTL--------------------------------------------------   94
OSyNE5              VF--FIVLTL--------------------------------------------------   94
MT325               VF--SIVVLGLKPELI----TNLI------------------------------------   95
ATCV1               VF--AIVISGFTFPW---------------------------------------------   82
OtV6                LI--LGILELISESKS----VANVVPTVMKK------MI---------------------   104
DishuiLV            VI--VGIVNLLSGGGI----SLKKNNAAMNT------ISN--TISNVPAPA---------   116
EsV1                MF--FVMLPVVAKALE----K---------------------------------------   124
BpV1                IL--VELATILSKMTK--------------------------------------------   83
MpV1                MI--GELAKILKIF----------------------------------------------   79


Vp2                 QNSHGLDKVFEQNRYVSDNFADGWITRGEDSSGLLIISLEAYCKETGKSTKRWIPTDTLK   203
Vp3                 KDKK--------------------------------------------------------   149
Moraxella           ----AMEPF-----SDKKD-----------------------------------------   158
Vp1                 PDAHGIDETLKQK-QFDVSQGKAIVTLGRDDGGRFIVATNTQT-DSGRNKLKWISYLKFD   203
VpVH7D              KDAHGIDTIIDQY-FDSNDKADRFVTLGQDDSGRYVVAINQLIVETQHTSMKWHTFINEK   189
VpGrn1              KDAHGIDTIIDQY-FDSNEKADRIVTLGQDDSGRYVVAINQLMVETQHTSMKWHTFINEK   182
VpValKK3            KDAHGIDTIIDQY-FDSNEKADRIVTLGQDDSGRYVVAINQLMVETQHTSMKWHTFINEK   209
VpST2               KDAHGIDTIIDQY-FDSNEKADRFVTLGQDDSGRYVVAINQLMVETQHTSMKWHTFINEK   143
Vpphipp2            KDAHGIDTMIDQY-FDSNDKAERFVTLGQDDSGRYVVAINQQIIETQKTSLKWHTFIKED   209
Vpnt1               KDAHGVDTFIDQY-YDSNDSADRIVTLGQDDSGRYVVAINQQMRDTQKTSMKWHTFILES   209
LpPLE2              --KRQLLRL---------------------------------------------------   259
Lactobacillus       --KRQLLRL---------------------------------------------------   259
MpMyrna             ------------------------------------------------------------   119
MpPhabba            ------------------------------------------------------------   118
SpBillNye           ------------------------------------------------------------   123
```

```
Acidobacterium      --E---------------------------------------------------------    128
Ptexasensis         ------------------------------------------------------------    122
Rheinheimera        --IRS-------------------------------------------------------    127
Ap                  --K---------------------------------------------------------    124
Ppventosus          ------------------------------------------------------------    117
YSLV                ------------------------------------------------------------    96
OLPV2               ------------------------------------------------------------    140
AaV1                ------------------------------------------------------------    156
TeV1                ------------------------------------------------------------    81
PBCV1               ------------------------------------------------------------    94
OSyNE5              ------------------------------------------------------------    94
MT325               ------------------------------------------------------------    95
ATCV1               ------------------------------------------------------------    82
OtV6                ------------------------------------------------------------    104
DishuiLV            ------------------------------------------------------------    116
EsV1                ------------------------------------------------------------    124
BpV1                ------------------------------------------------------------    83
MpV1                ------------------------------------------------------------    79


Vp2                 DQKYTYNRFINNMNEL---    219
Vp3                 -------------------    149
Moraxella           -------------------    158
Vp1                 DALDFYNDVAVELDAV---    219
VpVH7D              DAVKFFEETSELFHTELL-    207
VpGrn1              DAVKFFEETSELFHTELL-    200
VpValKK3            DAVKFFEETSELFHTELL-    227
VpST2               DAVKFFEETSELFHTELL-    161
Vpphipp2            D------------------    210
Vpnt1               DALKFYKDTSELFHTEIFS    228
LpPLE2              -------------------    259
Lactobacillus       -------------------    259
MpMyrna             -------------------    119
MpPhabba            -------------------    118
SpBillNye           -------------------    123
Acidobacterium      -------------------    128
```

```
Ptexasensis         -------------------   122
Rheinheimera        -------------------   127
Ap                  -------------------   124
Ppventosus          -------------------   117
YSLV                -------------------   96
OLPV2               -------------------   140
AaV1                -------------------   156
TeV1                -------------------   81
PBCV1               -------------------   94
OSyNE5              -------------------   94
MT325               -------------------   95
ATCV1               -------------------   82
OtV6                -------------------   104
DishuiLV            -------------------   116
EsV1                -------------------   124
BpV1                -------------------   83
MpV1                -------------------   79
```

.

Figure S1. Alignments of virus potassium channels. **A:** Alignment of known alga virus K^+ channels from the following viruses: Paramecium bursaria chlorella virus-1 (PBCV-1), Only Syngen chlorella virus (OSy-NE5), Ectocarpus siliculosus virus-1 (EsV-1), Chlorella Pbi virus MT325 (MT325), Acanthocystis turfacea virus-1 (ATCV-1), Bathycoccus sp. RCC1105 virus (BpV1), Micromonas sp. RCC1109 virus (MpV1), Ostreococcus tauri virus RT-2011 (OtV6), Dishui lake phycodnavirus 1 (DishuiLV), Yellowstone Lake phycodnavirus (YSLV), Aureococcus anophagefferens virus-1 (AaV1), Tetraselmis virus-1 (TeV-1), Organic Lake Phycodnavirus-2 (OLPV2). The conserved filter region is highlighted in yellow. Predicted TMs are underlined. Note that not all TMs were recognized by the algorithm even though the functionality of some of the channels was confirmed in experiments (e.g. K_{MpV1}). **B:** Alignment of all known viral potassium channel and closely related bacterial proteins. The alignment corresponds to the phylogenetic tree in Figure 2. Sequences were abbreviated as follows: Vibrio phage phi-pp2 (Vpphipp2), Vibrio phage nt-1 (Vpnt1), Vibrio phage ValKK3 (VpValKK3), Vibrio phage VH7D (VpVH7D), Vibrio phage phi-Grn1 (VpGrn1), Vibrio phage phi-ST2 (VpST2), Vibrio phage 1.081.O._10N.286.52.C2 (Vp1), Vibrio phage 1.084.O._10N.261.49.F5 (Vp2), Vibrio phage 2.275.O._10N.286.54.E11 (Vp3), *Pararheinheimera texasensis* (Ptexasensis), *Rheinheimera* sp. F8 (Rheinheimera), Acinetobacter phage (Ap), Pseudomonas phage ventosus (Ppventosus), Mycobacterium phage Myrna (MpMyrna), Mycobacterium phage Phabba (MpPhabba), Lactobacillus phage PLE2 (LpPLE2), Streptomyces phage BillNye (SpBillNye), Moraxella bacterium (Moraxella). In the alignment, identical amino acids are indicated by "*", conserved and semi-conserved amino acids are indicated by ":" and ".", respectively.

Figure S2

A

```
Vp2         ---MLNCITVIDSHGRKEYKTSFLLLVNIVTVLLNIYIGGLFLWHFEEGLQDSNINSLKE      57
Vp3         MKKYLHWCISTSSKMYKKYGTKYILILTGSITGLNVIIGALFLYLFEHNVDNALINSYSS      60
V1          -----MKFKSYDSFGVVEYNIGMIFAGLMALSFTIALTAASLLYRFESGADGSNINSAGD      55
VpVH7D      --------------------MLMIFGLMIVIALSIALTAANILFSFEIQNPDANITTPGD      40
VpValKK3    MKRFKKHIRKYDEHGVAQYNMLMIFGLMIVIALSIALTAANILFSFEIQNPDANIKTPGD      60
VpGrn1      ---------------------------MIVIALSIALTAANILFSFEIQNPDANITTPGD      33
Vphipp2     MKRFKKHIKKYDEHGVVQYNMLMIFALIVTISMSIALVASNILFAFELSASNANIKTPGD      60
Vnt1        MKRLKKHIRKYDEHGVVQYNMLLIFALIISISTSIAFIAANFLFYFEVQDPNANIRTYGD      60
                                                  .. :*: **    .: * :  .

V2          AMWAVFMTMTTIGFGDKYPITVEGYITTGVCFLLGAVNLGTLIKTASNLVKDK--NEVDN      115
V3          ALWMNWMVATTVGFGDYYPISTGGKIVTGISSAIGIGMFGLFTSVVSGILMEHTDDSIRN      120
V1          ALWTIWMSMSTIGFGDHYPVTVGGRVIVGSMFAVGATLIGLNIGIASAWVTSKFDKSVQN      115
VpVH7D      SFWTVWMAMSTIGFGDKYPVTTGGRYVIGCMFVVGTSLLGLTIGIINSWVVSKFDKSIQN      100
VpValKK3    SFWTVWMAMSTIGFGDKYPVTTGGRYVIGCMFVVGTSLLGLTIGIINSWVVSKFDKSIQN      120
VpGrn1      SFWTVWMAMSTIGFGDKYPVTTGGRYVIGCMFVVGTSLLGLTIGIINSWVVSKFDKSIQN      93
Vpphipp2    AFWTVWMAMSTIGFGDHYPVTTGGRFVIGSMFVVGASLIGLNIGIANAWITSKFDKSIQN      120
Vpnt1       AFWTVWMAMSTIGFGDHYPVTEGGRYTIGCMFVVGASLIGLNIGIVNGWISSKFDKSIQN      120
              *  :*  :*:**** **::  *    *    :*   :*      .  :  .:  ..: *

V2          RQIYSIITELLRSNQHIEQELNLTQEV--TQNSHGLDKVFEQNRYVSDNFADGWITRGED      173
V3          RELKEQNARIEIELNEVKKIL-----VSFTKDKK--------------------------      149
V1          RVLKLQSDTLIQKIDRLETFLNVDQTVYFGPDAHGIDETLKQK-QFDVSQGKAIVTLGRD      174
VpVH7D      RVLKSQNDALLHKLDRLETHLGIDQEVRFDKDAHGIDTIIDQY-FDSNDKADRFVTLGQD      159
VpValKK3    RVLKSQNDALLHKLDRLETHLGIDQEVRFDKDAHGIDTIIDQY-FDSNEKADRIVTLGQD      179
VpGrn1      RVLKSQNDALLHKLDRLETHLGIEQEVRFDKDAHGIDTIIDQY-FDSNEKADRIVTLGQD      152
Vphipp2     RVLKSQNDALLQKLDRLETHLGIDQEVLFSKDAHGIDTMIDQY-FDSNDKAERFVTLGQD      179
Vpnt1       RVLKSQNDALLQKLDRLETFLGVNQEVLFDKDAHGVDTFIDQY-YDSNDSADRIVTLGQD      179
              :      :  . :.::  *     *    : :
```

```
V2          SSGLLIISLEAYCKETGKSTKRWIPTDTLKDQKYTYNRFINNMNEL---    219
V3          ------------------------------------------------    149
V1          DGGRFIVATNTQT-DSGRNKLKWISYLKFDDALDFYNDVAVELDAV---    219
VpVH7D      DSGRYVVAINQLIVETQHTSMKWHTFINEKDAVKFFEETSELFHTELL-    207
VpValKK3    DSGRYVVAINQLMVETQHTSMKWHTFINEKDAVKFFEETSELFHTELL-    227
VpGrn1      DSGRYVVAINQLMVETQHTSMKWHTFINEKDAVKFFEETSELFHTELL-    200
Vpphipp2    DSGRYVVAINQQIIETQKTSLKWHTFIKEDDATKFYKDTSELFHTELFI    228
Vpnt1       DSGRYVVAINQQMRDTQKTSMKWHTFILESDALKFYKDTSELFHTEIFS    228
```

B

```
Vpphipp2    MKRFK--KHIKKYDEHGVVQYNMLMIFAL-IVTISMSIALVASNILFAFELSASNANIKT   57
Moraxella   MSRFTLSKRIYRTDEFEVRNYNMLFLTFVAVVILYLSIAVLAF-LLLWAESGAQGANIET   59
            *.**.  *:* : **. * :****::  : :* : :***::*  :*:  * .*..***:*

Vpphipp2    PGDAFWTVWMAMSTIGFGDHYPVTTGGRFVIGSMFVVGASLIGLNIGIANAWITSKFDKS   117
Moraxella   YGDAFWTLQMSASTIGFGDYYPVTLGGRTIVAAMFYIGVGIVGFIGAQVADRFLGFADTN   119
             ******: *: *******:**** *** ::.:** :*..::*:  . .   : .  *..

Vpphipp2    IQNRVLKSQNDALLQKLDRLETHLGIDQEVLFSKDAHGIDTMIDQYFDSNDKAERFVTLG   177
Moraxella   VKNRELRKQNEQILEHNKVLEKKLDKL------------LSAMEPFSDKKD---------   158
            ::** *:.**: :*:: . **.:*.               : :: : *.:*

Vphipp2     QDDSGRYVVAINQQIIETQKTSLKWHTFIKEDDATKFYKDTSELFHTELFI          228
Moraxella   ---------------------------------------------------          158
```

Figure S2. Putative potassium channels from vibrio phage. **A:** Alignment of vibrio phage potassium channel sequences. The conserved signature motif (T/S)T(I/V)GFGD is highlighted in yellow. The TM domains as predicted by TMHMM are underlined. **B:** Alignment of the vibrio phage potassium channel versus the most similar protein from a bacterium of the Moraxellaceae family. Viruses: Vibrio phage phi-pp2 (Vpphipp2), Vibrio phage nt-1 (Vpnt1), Vibrio phage ValKK3 (VpValKK3), Vibrio phage VH7D (VpVH7D), Vibrio phage phi-Grn1 (VpGrn1), Vibrio phage phi-ST2 (VpST2), Vibrio phage 1.081.O._10N.286.52.C2 (Vp1), Vibrio phage 1.084.O._10N.261.49.F5 (Vp2), and Vibrio phage 2.275.O._10N.286.54.E11 (Vp3). In the alignment, identical amino acids are indicated by "*", conserved and semi-conserved amino acids are indicated by ":" and ".", respectively.

Figure S3

A

```
AngHV-1                   ------------------------------------------------------------
Pundamilia nyererei       MFFIALLWSLSLTAVGQQEDNEWLDPYDMLNYDASTKTMRKPTEPASYDNVGTKRREYNQ

AngHV-1                   ------------------------------------------------------------
Pundamilia nyererei       DSSQTELTSCKEQVEDLQKQNEDQKKTIKLISQQPTCSPVFKRFLSRLLKEIQRVGVPSD


AngHV-1                   ---------MKMNR-ILVLLLLFWGTRAEWDVTSYTAKLGLVKGCKCRPEEVKEVKKVWR
Pundamilia nyererei       STDVFFDAKVKLSKQAMTEIQTLLEGEDRWRTGALDNAISQIL-VDLKPH--DYEAWKWR
                                   :*:.:  :. :  :   . .* . :    :. :   . :*.  .     **

AngHV-1                   FEDTFGVEIDTVLQICSCVLVIVMIVCGELWSTVSWFLQLERAFIICLFVSFIWNWIYLY
Pundamilia nyererei       FEDTFGVELDTVLKIGSFVLIIVAIISTELWTTISWFVQFRRLFAVCFFVSIIWNWFYLY
                          ********:****:* * **:** *:. ***:*:***:*:.* * :*:***:****:***

AngHV-1                   KAAYAEHQANMIKLDGVAKRCAN---ADFMSTLKDWFRSTWTLQDDPCKKYHEVLIINPV
Pundamilia nyererei       KIAFAEHQNNIVKMEGFNAKCTGVKKIDWSDSLKEWFRSTWTLQDDPCKRYYEVLMVNPI
                          * *:**** *::*::*.  :*:.    *: .:**:**************:*:***::**:

AngHV-1                   LLVPPTKAISVTFVTFVTEPLKHFGHGIGEFIKALLKDLPITLQVPVWGDHFSSRCRVYV
Pundamilia nyererei       LLVPPTKAISVTITTFITEPLKHFGQGISEFLRALLKDLPVTLQIPVLLTIVIAVVVFMY
                          ************:.**:********:**.**::*******:***:**    . :   .

AngHV-1                   ---RNLFCGGWPLISLRTTHTTHTTHE------DR-----RWKHFWK-------CGLDFR
Pundamilia nyererei       GSVQAAFQHGIM-APLRRPRRDPPPPELEQPQAQPRLRIEDRNHFAGGDAPQQQHALRYR
                             :  *  *     **  :      *      :        :**          .* :*

AngHV-1                   GYLN---------------RVA------EFPAAERE------------------------
Pundamilia nyererei       ADEARLNRNDLHQRRPNRQRVETLGTAEDEPDAERAKEVPEADQNLSVEPDSENQRGAEE
                          .                  **       : *  ***
```

```
AngHV-1               ------------------------------------------------------------
Pundamilia nyererei   RPPAAKDNSVGENKAKSEPKAARSDSSESKNKTSKGNENLSQDQPDRDVALLRPELPDST
```

B

```
AngHV-1               ------------------------------------------------------------
Homo sapiens          MLCSLLLCECLLLVAGYAHDDDWIDPTDMLNYDAASGTMRKSQAKYGISGEKDVSPDLSC

AngHV-1               --------MKMNRILVLLL-LFWGTRAEWDV-------------TSYTAKLGLVKGCKCR
Homo sapiens          ADEISECYHKLDSLTYKIDECEKKKREDYESQSNPVFRRYLNKILIEAGKLGLFHD----
                               *:: :   :      .* :::                 :.****.:.

AngHV-1               PEEVKEVKKVWRFEDTFGVEIDTVLQICSCVLVIVMIVCGELWSTVSWFLQLERAFIICL
Homo sapiens          -----FETWKWRFEDSFGVDPYNVLMVLLCLLCIVVLVATELWTYVRWYTQLRRVLIISF
                             .  *****:***:  .** :  *:* **::*. ***: * *: **.*.:**.:

AngHV-1               FVSFIWNWIYLYKAAYAEHQANMIKLDGVAKRCAN-ADFMSTLKDWFRSTWTLQDDPCKK
Homo sapiens          LFSLGWNWMYLYKLAFAQHQAEVAKMEPLNNVCAKKMDWTGSIWEWFRSSWTYKDDPCQK
                      :.*: ***:**** *:*:***:: *:: : : **:  *: .:: :****:** :****:*

AngHV-1               YHEVLIINPVLLVPPTKAISVTFVTFVTEPLKHFGHGIGEFIKALLKDLPITLQVPVWGD
Homo sapiens          YYELLLVNPIWLVPPTKALAVTFTTFVTEPLKHIGKGTGEFIKALMKEIPALLHLPVLII
                      *:*:*::**: *******::***.*********:*:* *******:*::*  *::**

AngHV-1               HFSSRCRVYYVRNLFCGGW--PLISLRTTHTTH------TTHEDRRWKH---------FWK
Homo sapiens          MALA------ILSFCYGAGKSVHVLRHIGGPESEPPQALRPRDRRRQEEIDYRPDGGAGD
                         :         ** *    :  **     .         .*** :.           .

AngHV-1               CGLDFRGYLNRVAEFPAAERE---------------------------------------
Homo sapiens          ADFHYRGQMGPTEQGPYAKTYEGRREILRERDVDLRFQTGNKSPEVLRAFDVPDAEAREH
                      ..:.:** :. . : * *:
```

```
AngHV-1          ------------------------------------------------------
Homo sapiens     PTVVPSHKSPVLDTKPKETGGILGEGTPKESSTESSQSAKPVSGQDTSGNTEGSPAAEKA

AngHV-1          --------------------------------
Homo sapiens     QLKSEAAGSPDQGSTYSPARGVAGPRGQDPVSSPCG
```

Figure S3. Putative CLIC-like chloride channels from AngHV-1. Alignments of the CLIC-like chloride channel from Anguillid herpesvirus-1 (AngHV-1) versus the chloride channel from the fish *Pundamilia nyererei* (**A**) versus the human homolog (**B**). Predicted TM domains are underlined; conserved regions are shown in yellow. In the alignment, identical amino acids are indicated by "*", conserved and semi-conserved amino acids are indicated by ":" and ".", respectively.

Figure S4

A

```
PBCV-1      MEDIDNIYDCTPSTLTNLTACFIPSSEAWCNIGDTCSVSNIRYDPKCNFFGYADQYLTHI   60
OSy-NE5     MEDGDDLYDCTTAIITNITACFIPSSEAWCNIGDTCEVSTSRYDPKCNFYGYADQYLTHL   60
            *** *::**** : :**:******************.**. ********:*********:

PBCV-1      IPRTFKCFNVIRQCVTSYTDAERQGCDILYGEPNPLAYTNKSNYWAESVLYDVDSPAILF   120
OSy-NE5     IPRTFKCFNVTRRCVTGYNDAEYQGCDMLYGEQTPLAYVNQSDYWSGSVLYDVDTPAILF   120
            ********** *:***.*.*** ****:**** .****.*:*:**: *******:*****

PBCV-1      NPDKSYIPWDFLKPFSWELWAMLIIVMCIITPLVTSLIEYDTGETVVGNFIKYLPDSIHA   180
OSy-NE5     NPEHSYTPWDFLNPFSWQLWLMIFITMFIITPLVTSFVEYDTGETILGNFLKYIPDSIHA   180
            **::** *****:****:** *::*.* ********::*******::***:**:******

PBCV-1      HLGIDLISSESMTNNTSYTLAVFINIFAIIMLALYSSNLVAYVLYKNYSLSSLPYSYKPT   240
OSy-NE5     HLGVDLISSESMTNNTSYTHAVFINIFAIVIFMLYSSNLTAYVLYKNYSESTLINSYRPS   240
            ***:*************** *********::: ******.********* *:*  **:*:

PBCV-1      WDIFIDNTISNSFSELPNDLLYIDSSKIPEIIDSSEFDAIIGQRTFLQIFKDCSSELTII   300
OSy-NE5     WNIFVDNSISEAYSSLPNDLFYIDSSKIPVLIESSDFDAIIGQRTFLQIYKSCASELTII   300
            *:**:**:**:::*.*****:******** :*:**:*************:*.*:******

PBCV-1      HGPGIYKYVNLATKAGMLNIRRLIAQIQMASVNRQFTSPTCTEQPQSINLNSMYGVFLLF   360
OSy-NE5     QGPGIYKYVNLATKLSMKHIRGLTAQIQMASVNRQFTSPTCSDKPQSIELSSMYGVFLLF   360
            :************* .* :** * *****************:::****:*.*********

PBCV-1      FIPMTLLFLITIIRFT-LKRKFSADNSMVIPKTHSPDDLEKYEAEGSSESVV         411
OSy-NE5     FIPVSLLFTGSIIRSMILKGRKSSVNSMMITHTRSPDESVNMQEVV------         406
            ***::***  :***   ** : *: ***:* :*:***:  : :
```

B

```
PBCV-1                  -------------------------MEDID--------NIYDCTPSTLTNL---------   18
Cricetulus griseus      MCPETEEQEDDHLSIVTLEEAPFVIVESVDPLSGTCMRNTVPCQKRIISENKTDEEPGYI   60
                                                 :*.:*        *   *    :::

PBCV-1                  TAC-----------------------FI-------PSSEAWCNIGDTCSVSNIRYDPKCN   48
Cricetulus griseus      KKCCKGFCIDILKKISKSVKFTYDLYLVTNGKHGKKINGTWNGMIGEV---VMKRAPLCY   117
                        . *                       ::         . :* .: .      ::  * *

PBCV-1                  FFGY--ADQYLTHIIPRTFKCFNVIRQCVTSYTDAERQGCDILYGEPNPLAYTN-KSNYW   105
Cricetulus griseus      NVLTVASYCFLQVVMKRAPACYNILTVASYCFLQVVMKRAYMAVG---SLTINEERSEVV   174
                         .    :  :*  :: *:  *:*::  .  .: :.  : . :  *    *: .: :*:

PBCV-1                  AESV-LYDVDSPAILFNPDKSYIPWDFLKPFSWELWAMLIIVMCIITPLVTSLIEYDTGE   164
Cricetulus griseus      DFSVPFIETGISVMVSRSNGTVSPSAFLEPFSADVWVMMFVMLLIVSAVAVFVFEYFSPV   234
                          ** : :..  .:: . : :  *  **:*** ::*.*::::: *:: :.. ::** :

PBCV-1                  TVVGNFIKYLPDSIH--AHLGIDLISSESMT---NNTSYTLAVFINIFAIIMLALYSSNL   219
Cricetulus griseus      G----YNRCLADGRAIWLLWGLVFNNSVPVQNPKGTTSKIMVSVWAFFAVIFLASYTANL   290
                             : : * *.       *: : .*  :    ..**  :. .  :**:*:** *::**

PBCV-1                  VAYVLYKNYSLSSL-------PYSYKPTWDI------FIDNTISNSFSELPNDLLYIDSS   266
Cricetulus griseus      AAFMIQEEYVDQVSGLSDKKRPNDFSPPFRFGTVPNGSTERNIRNNYAEMHAYMGKFNQR   350
                        .*::: ::*  .         * .:.* : :        :..* *.::*:   :  ::.

PBCV-1                  KIPE---IIDSSEFDAIIGQRTFLQIF------------------KDCSSELTIIHGPGI   305
Cricetulus griseus      GVDDALLSLKTGKLDAFIYDAAVLNYMAGRDEGCKLVTIGSGKVFASTGYGIAIQKDSGW   410
                         : :    :.:.::**:* : :.*: :                   . .  ::* :. *

PBCV-1                  YKYVNLATKAGMLNIRRLIAQIQMASVNRQFTSPTCTEQ-----PQSINLNSMYGVFLLF   360
Cricetulus griseus      KRQVD-------LAILQLFGDGEMEELEALWLTGICHNEKNEVMSSQLDIDNMAGVFYML   463
                         : *:       * * :*:.: :* .::  : :  * ::      ..::::.* *** ::
```

```
PBCV-1                FIPMTLLFLITIIRF----TLKRKF----SADNSMVIPKTHS------------------   394
Cricetulus griseus    GAAMALSLITFICEHLFYWQFRHCFMGVCSGKPGMVFSISRGIYSCIHGVAIEERQSVMN   523
                         *:* ::  *  ..     ::: *    *.. .**:  ::.

PBCV-1                -PD------------------DLEKY-EAEGSSESVV-----------------------   411
Cricetulus griseus    SPTATMNNTHSNILRLLRTAKNMANLSGVNGSPQSALDFIRRESSVYDISEHRRSFTHSD   583
                       *                   :: :   .:** :*.:

PBCV-1                ------------------------------------------------------------   411
Cricetulus griseus    CKSYNNPPCEENLFSDYISEVERTFGNLQLKDSNVYQDHYHHHHRPHSIGSTSSIDGLYD   643

PBCV-1                ------------------------------------------------------------   411
Cricetulus griseus    CDNPPFTTQPRSISKKPLDIGLPSSKHSQLSDLYGKFSFKSDRYSGHDDLIRSDVSDIST   703

PBCV-1                ------------------------------------------------------------   411
Cricetulus griseus    HTVTYGNIEGNAAKRRKQQYKDSLKKRPASAKSRREFDEIELAYRRRPPRSPDHKRYFRD   763

PBCV-1                ------------------------------------------------------------   411
Cricetulus griseus    KEGLRDFYLDQFRTKENSPHWEHVDLTDIYKERSDDFKRDSVSGGGPCTNRSHLKHGSGD   823

PBCV-1                ------------------------------------------------------------   411
Cricetulus griseus    KHGVVGGVPAPWEKNLTNVDWEDRSGGNFCRSCPSKLHNYSSTVAGQNSGRQACIRCEAC   883

PBCV-1                ------------------------------------------------------------   411
Cricetulus griseus    KKAGNLYDISEDNSLQELDQPAAPVAVTSNASTTKYPQSPTNSKAQKKNRNKLRRQHSYD   943
```

```
PBCV-1                  ------------------------------------------------------------        411
Cricetulus griseus      TFVDLQKEEAALAPRSVSLKDKGRFLDGSPYAHMFEMPAGESSFANNKSSVPTAGHHHNN        1003

PBCV-1                  ------------------------------------------------------------        411
Cricetulus griseus      PGGGYMLSKSLYPDRVTQNPFIPTFGDDQCLLHGSKSYFFRQPTVAGASKTRPDFRALVT        1063

PBCV-1                  ----------------------------------------------------------          411
Cricetulus griseus      NKPVVSALHGAVPGRFQKDICIGNQSNPCVPNNKNPRAFNGSSNGHVYEKLSSIESDV          1121
```

Figure S4. Putative glutamate receptor-like channels from Phycodnaviridae. **A:** Alignment of the putative glutamate receptors-like channels from viruses PBCV-1 and OSy-NE5. **B:** Alignment of the putative glutamate receptor from chlorovirus PBCV-1 versus the homologous protein from *Cricetulus griseus* (chinese hamster). Predicted TM domains are underlined. In the alignment, identical amino acids are indicated by "*", conserved and semi-conserved amino acids are indicated by ":" and ".", respectively.

Figure S5

```
AaV          ---MRET---FTDLYNSPNFIYIR----------YISAIIIVLLTFPISSILTQKLQDTD      44
TeV1         MGAKKPKYKNFVHWLDEKSVISVAVAFSVSMAVNRFMQTLIDNLVIAIISKTTGAE----      56
CroV         ---MSQIYVDFKDFLKDNDIIVTIIATIVSSNISMLSKSFMKNLVMPIINIDLNNDGIPD      57
                       * .  .. ..*              :   ::  *.: * .

AaV          YKNEDFDKFFQTGLSIL----LIRFATMFVLILIALKVANVNNLVIASYAGILLIAVPAA      100
TeV1         ---DLEWEI-TKELSIKYGKIIVESINLMIILYLSYLIIK--------------------      92
CroV         RQNLDNWVIHMKGVDLKIGQFLLTFIEFFLILIIIYLINK--------------------      97
                     :  . :.:     ::    ::::: :   : :

AaV          MSTQISNYISGLLLIAFDRITLNDYIIIDDFEGRIKKLNLFSIEVKDEFTKKTRFIPNAD      160
TeV1         ------------------------------------------------------------      92
CroV         ------------------------------------------------------------      97

AaV          FWTKSFINVSKNTTAVAKIEITVASDNDFDEIEDKILNIIDNNFEGIDASKTRIRYDHSI      220
TeV1         ------------------------------------------------------------      92
CroV         ------------------------------------------------------------      97

AaV          WGVKLSIAVEVPSKKYFEYKMLLLRVIRKKISEDEDINFVM                         261
TeV1         -----------ASNRYLGWT---------------------                         101
CroV         -----------LSKI--------------------------                         101
                         *:
```

Figure S5. Putative mechanosensitive-like channels from large DNA viruses. Alignment of putative mechanosensitive ion channels from Aureococcus anophagefferens virus (AaV), Tetraselmis virus-1 (TeV1), and Cafeteria roenbergensis virus (CroV). Predicted TM domains are underlined. In the alignment, identical amino acids are indicated by "*", conserved and semi-conserved amino acids are indicated by ":" and ".", respectively.

Figure S6

```
KNV1        MLEVLFGISNTNFSNNEVKRTKHSIFWIPLLTIYKYFFWNYENIYFLFLSLFQLSTLYFL      60
CTV1        ----MEGDNSI--YLTRFKFSGLILYVCHIIYGYINYFFDFINIFCLLISFFQIYDY---      51
                : * ..     ...* :   ::   ::  *  :*::: **: *::*:**:

KNV1        PKEWSPTGPYSTAIPLGICILAEIITHMITWYNDWITDYKENNKEYECLDHSYQLIKKKN      120
CTV1        ----R---DFRSFIPLSIFS--TLY---SVYYTYTVSKLI---REQNSI-NSEMFMRKPI      95
                     : : ***.*     :     .:*.  ::.     :* :.: :*  :::*

KNV1        RNIYPGDIIHLEKEDICPIDGILIDTTNNEKYSKISLALLTGESNINYVIKPAKLFKLQD      180
CTV1        NQIKRGDILELSYQDKIPADILILTDDFHVSTNELE---LSGE---NIVLNKKALF--ED      147
            .:*  ***:.*. :*  * * :::    : . .::.   *:**   * *::   **  :*

KNV1        YKDYKIN-----ISNYHQNNFNNIEGKLLNGKEEHNIQGENFVVGGSIIKSDDIYLWVIG      235
CTV1        YDNNQLLKSNICINQKKNNGFIEYDNKK------YKYDENNIVFRGTKMLDGKLKGLVVE      201
            *.: ::      *.: ::*.* : :.*       :: : :*:*. *: : ...:   *:

KNV1        CGRDKKSYLKKS-VKNDRKKNRIDTFVGNYMINVNAILLLLLI--LITTTIKLVNSFSFG      292
CTV1        IGNDCMIYNIDNRVLKD--KTWLYKKVNN--ITFNNLYYLLIISAFIAGALK--YVYPQR      255
             *.*   *  .. * :*  *. : . *.*  *..* :  **:*  :*: ::*    :

KNV1        NIIFYMIQNWILFNGIMPFSVKIFLL-----LARNLQTGIHNYYHKSITINNSLLIDDIG      347
CTV1        KFIFLVKTTVLLLNTVVPLSLQSFYNACTWILSRKIQTEN------NVTINSHGINCFEN      309
            ::** :  . :*:* ::*:*:: *       *:*::**        .:***.  :    .

KNV1        KINKILTDKTGTLTKNELEFSKLLESW-KNDIIDVET-----YQQNYYDIDLNFHKCIGL      401
CTV1        NPKYVVTDKTGTITKNQLKLIQVINISNKNNIIEKNDITNSSNINNFFDI----MSCTLI      365
            : : ::******:***:*:: ::::   **:**: :        :*::**     .*  :

KNV1        CIHQTEENYSTPED-KTLRYRYQYLNNRINQTSQI-ITLTINNNNYDYKY---------I      450
CTV1        NTHSTTKQLLKNDEMEYLLLEWCCKNNKIKIMNNNYNEINKNLSFYKYKFNNKEEKINKI      425
              *.* ::  . :: : *  .:   **:*:  .:    :. * . *.**:         *

KNV1        EIGGLDFTFERRLSSKIVKDISTDSYYIYCKGAMDVIGKKIKTDYKTE-LKRLDQMVSQK      509
```

```
CTV1      IYKGFDYKLGIK----YCITEKDDIYTLNIQGTPEMINLYTNKNLINEGEKLLDEVSNNS      481
             *:*:.:  :         . * * :  :*: ::*.   :.:  .*  * **:: .:.

KNV1      YPELRLLACAFRKIDKNELDMALHESTNKSQIVTLLENDLHLLGIIGIKDNLQEGVKETI      569
CTV1      Y---RRIICYAKKNITSDTYLDIKNNCN---IN-EYLKDFEYANIYVFEDQVMDDLANHF      534
          *   * : *  :*   ..:  : :::. *   *     :*:.  .*  ::*:: :.: : :

KNV1      EQFNHYGLFSCLLTGDRKITALAIAKEAGIIDHENTICDFTQEMLDKDITNLHKK-TILF      628
CTV1      DKLMKNGKHITILTGDRHSSSVEVGKILGMCDDMVLID-KK-----EDMNNIDKTVSLSV      588
          ::: : * .  :*****: ::: :.*  *: *.   *   .     :*:.*:.*. :: .

KNV1      GGALFDIVSQNVKYSESFYDKLALSRNFIGYNLIPEHKKKLTNILE--NKNIKTLTVGDG      686
CTV1      NGKIFNELIREEK----FKNIILNTNKIIVYRATPEIKEKYIQYLKFADEKNQVMMIGDG      644
          .* :*: : :: *    * : :  :.::* *.  ** *:*  : *:  ::: :.: :***

KNV1      FNDIGMFHTSSMSIAIKGNGFVESN-ADFTIKEFKQLKHLFD---MSLKYYSKNAQLVNL      742
CTV1      SNDISAIMRADVGVAVKGESNQIQNISDVVIDSWCKIPELLKNFSYKKEIIEHNVKWVLT      704
           ***. :  :.:.:*:**:.   .* :*..*..: :: .*:.    . :  .:*.: *

KNV1      TFLRASAVIMSIMTYSLIYYNQ---TTSLFNGFVIQAFNFAWTILGVGYYTLKQRNLPHQ      799
CTV1      KHIMTATILMTMLLI--SNYKEIRDPTNPFHMLILNCLLFVCMSMFSFNNNLRIINNPIN      762
          ..: :::::*:::      *::    *. *: ::::.: *.   :     .*:  * * :

KNV1      DQDYYQNKHLVLTNYKNTSIWNGAGIIFGIVLTLMNYYWFRESKYFGDICGLMLVMILNG      859
CTV1      EKDYKTHI--------------HKGIIVGMIIGTIVFTIFSV------NIGIIIAI----      798
          ::**  :                 ***.*:::  : :  *          *:::.:

KNV1      KLILNNKLDLWGIGLSLMGIVNFMGYMMYMGSLYDVIITLLTTSKYYWLGVFGMYFGINL      919
CTV1      -------------------I----AKFIYLSLIL--------------------------      809
                             *    . ::*:. :

KNV1      FIF 922
CTV1      --- 809
```

Figure S6. Giant virus ATPases. Alignment of Klosneuvirus-1 ATPase vs. Catovirus-1 ATPase. Predicted TMs are underlined. In the alignment, identical amino acids are indicated by "*", conserved and semi-conserved amino acids are indicated by ":" and ".", respectively.

Figure S7

```
ACEV                    --MLLPILWKHLITRFNKISSIILTVSEILIPILTIVLVLYYKSKLGNIDKIYINSTTYN 58
Onthophagus taurus      MAQLWTVIWEHCKIRKR---HYVLTLLELCIPIIIFVLVAFGRSQLPSFNKEFIPVTKN- 56
                           *  ::*:*   * .     :**: *: ***: :*** : :*:* .::* :*  *.

ACEV                    DKLLCTDLLTELRIGNTKILYSPSTIYNDDLINRVKIKLNLYSNDIKYFQNEKKLIEYYN 118
Onthophagus taurus      -YITPDSELSHINIVDTKLFYSPKTAFTADLARQVQLKLYMFNENIKGFDSKEDVLRYYN 115
                          :   . *:.:.* :**::***.* :. ** .:*::** ::.::** *:.::.::.***

ACEV                    TNKFDDLSTIVVI--IFNNTDKNLKYDIRIYENGIFWNTDKLFTNIYNY-DLAASNTYIT 175
Onthophagus taurus      EMGGDGITVIVNFDDVDQKIPTTLKYDIQMYEEGIDWETEKLFRKDIRYYPGYGSSHYLG 175
                            *.::.** :  : ::  ..*****::**:** *:*:*** :  .*    .*. *:

ACEV                    KSFNAIQLAIDISYIEMVINNTVDFQLTYQEFPYPPYLKTDNITISFRYILPFIIILGFI 235
Onthophagus taurus      NQFLSFQWALDTAYIEMLTKSPLNITINFEEFPYPAHWTDNGMNDIFVHLLPLLTMLSFI 235
                        :.* ::* *:* :****: :. ::: :.::***** : . :.:.  * ::**:: :*.**

ACEV                    LICPITLKTILMEKSSGLKELLKINGVKSVTLWIGWFLNQIIINIVSVIFICIILTTIRF 295
Onthophagus taurus      FLFPAILKMIVEEKSSGMKQLMKINGLKSSIYWLGWFLNASIPCIISAIVVAILLTQRLF 295
                        :: *  ** *: *****:*:*:****:**   *:*****  *  *:*.*.:.*:**   *

ACEV                    SDKYRIIEHCNPLILIIFLLLYCSSTITFFFAFSTLFNKIILSMIMGILLWISIYCIFII 355
Onthophagus taurus      SAEYPSLQHASMPQLALLILLYCLATISFIFAFSIFFKDATLAMITGITVWFLIYIIYAS 355
                        * :*  ::*..   * :::**** :**:*:**** :*:.  *:** ** :*: ** *:

ACEV                    NVENINDTI--ITNILLLFPPCTLYYGFDVLSLFEMRNTNINFDNLFESPAKGLSIGLVI 413
Onthophagus taurus      TVGNVDTLPLYLKYFASLIPTIALYNGFQVISVAEIRASPVGFTEQPRGCSDDMTMQTTF 415
                        .* *::     :. :  *:*  :** **:*:*: *:* : :.* :  .. :..:::  .:

ACEV                    IILFIDSILYFLIALYLDKLWPGKYGTSSSINMF-----NFRKKKTN--------QFLLQ 460
Onthophagus taurus      IMLIIDTLLYLTIAICCETDPHGKYKIANFCRNVKNKIDKFKKSDNTGVNNEEEHLIGIE 475
```

```
                        *:*:**::**: **:  :.   ***  :.  . .     :*:*....         : ::

ACEV                    D--LNSVIQLKNVSKIYNKKIKVIDDISFDIKKNKISILLGNNGAGKSTLMSMIMRMTSI 518
Onthophagus taurus      DPNDEVLLEIKNLTKKYNDL-VAVNKLNMKIYKDNLTVILGHNGAGKTTTLSILAGLEKE 534
                        *   : ::::**::* **.   .::.:.:.* *::::::**:*****:* :*::  : .

ACEV                    TSGHITG--------IERNKIAYCPQDNLLYKELTVIQHLILCGLLHGLNKNESKQKGEY 570
Onthophagus taurus      TSGELTIAEIENVNLKDRQLVSICPQENMLFTELTVEEHLVLFGKLKGLSDEDIKQNVKN 594
                        ***.:*          :*: :: ***:*:*:.**** :**:* * *:**..:: **: :

ACEV                    LLKNVLNMIDKKNLYPNELSGGMKRKLSLGIALMTNPDVLILDEPTSSIDIEGRQEIYNL 630
Onthophagus taurus      LLENKLNLANKKDDLVKNLSGGMKRKLSIGIAIMGTPKVVILDEPTAGIDPEGRREIWDL 654
                        **:* **: :**:   ::**********:***:* .*.*:******:.** ***:**::*

ACEV                    ILSLNK--TVLISTHLLEEAEYLADYIIIINSGKLLYYGNIIDLKNKYDIGYICTIKNKS 688
Onthophagus taurus      ILECKKDSNIILSTHLMEEAEELADRVAILGNGTLERYGKPMDLKQKLGVGYRIQLFYED 714
                        **. :*  .:::****:**** *** : *:..*.*  **: :***:* .:**   :  :.

ACEV                    IDENKI-KEIV-----GHIKIKDDNIICNI--DKTNKNKVPLLLKYLD----EL-NIEYL 735
Onthophagus taurus      PTKEKVIKTFVEENIDAKINPDNNKLVLEFCITPEKKCMIPKFKEVLNSRKGELSIKDYK 774
                          ::*: * :*     .:*: .::::: ::     :*  :* : : *:    **   :*

ACEV                    FSSISLNDLYIKVQNKE---II-NNNNQYYINHI--NQSLRIKINYYFNK------RKLF 783
Onthophagus taurus      IRSVTLEDVFLRSLENHDREIRLTINRQESINSQGTHKNLTCFPNNLWHKFVLIAEKKMK 834
                        : *::*:*::::  ::.   *  . *.*  **    ::.*    *  ::*      :*:

ACEV                    FIRKIK-QYLYIYIISLCIIGLSIYLSNFGYVNLIDDGNKIKLDLDVYGHTDIYYSLVGQ 842
Onthophagus taurus      ILRAMPIPWITMIALFYSLFALSFAMNDSGHKHTDRDGKTLQLNLDSYGCTKVFYEMKND 894
                        ::* :   :: :  :  .::.**: :.: *: :   **:.::*:** ** *.::*.: .:

ACEV                    DS------INIITHTSNSLIKVDNVNKSIIDVGINNIVHYIYKLIIAFEFNFTN--NSIN 894
Onthophagus taurus      DANLERIFVDLIDSTISNSHKVDDISEAVISVGENNIVKYRQKLIVGAKMNFDESSREIT 954
                        *:      :::*  * ..  ***::.:::*.** ****:*  ***:. ::** :  ..*.
```

```
ACEV                    IMYSNKAYHSMPIAINVFSNTLLNYYTNDNYSIYLTHTPLHSNNKQIEKIQIGEYSNLFI 954
Onthophagus taurus      VFYGSQPIHGAPISFNLLSNVLAKYLVGENHEIIASHTPLRSVKSTTRQTNVYVADYIIS 1014
                        ::*..:  *. **::*::**.* :* ..:*:.*  :****:* :.  .: ::   . ::

ACEV                    WSILLPIGPLILISTSLLLPAIDYVKNIKTLERLCNIKAYQYWLFNYVFDITCYIASISI 1014
Onthophagus taurus      WMILLSNACLFSVIMFSALPTLEKNSFIKTLSRIYNLNSICYWLHNYITDVIIYLVMIIP 1074
                        * ***  . *: :     **:::  . ****.*: *:::  ***.**: *:  *:. *

ACEV                    ILIIIYIMPIH---YLDVYTIIQLGGLFILYGIVFIPQSYIFTYMSTVENTYFNFLIV-H 1070
Onthophagus taurus      PIAVLYVVSKLDWALLQIDGYCYLVIILIWYGITMIPHTYLSSFYSKSVSNAIALLLIPN 1134
                         : ::*::       *::     *  ::* ***.:**::*: :: *.  .. : :*:: :

ACEV                    IFCSPLISVILII-----IDKEYIIILILLLSPHTSLCYYLASISIKLIRYHNWNIKSYE 1125
Onthophagus taurus      MLSSIPVGVILSLQSSGSVAPQIFLYLFALLDPHIILTYTLSIFCDKMIKNHNWDMKTPS 1194
                        ::.*  :.*** :     :  : :: *: **.**  * * *: :. *:*: ***::*: .

ACEV                    HKKAICSITDHVCCNLNSLECNDFKIFIDYKLIFCS-IISLCISIFILLSMNYITRNISK 1184
Onthophagus taurus      QKEYICNETPLPCCDPDSVECAEDRAYIQYPIYLMISVVSALIVMFIIV---WLDK---- 1247
                        :*: **. *   **: :*:** : : :*:* : :   ::*  * :**::   :: :

ACEV                    LLLYKQFNKTNTQSTSNNNILYINNVSKGYWKWPCNVLKVINDITIVINNNICFGLLGTN 1244
Onthophagus taurus      EKSYKKNHEEGVTIG--AKEVQKHYNIRTLRSFKKTTVKAVDDLSFTVSKGECYGLLGVN 1305
                           **: :: ..      : :  :   :   .:  ..:*.::*:::.:.:. *:****.*

ACEV                    GAGKTTMFKMMTNEIEPTIGKIK--INGTIGYCPQQDSLIDEFTGKELLYLYGKLRGITN 1302
Onthophagus taurus      GAGKSTSFKILTNQVRPDKGEIFRRQNSLIGYCPQEHALLNFFTGRELLKYFGKLRNPSI 1365
                        ****:* **::**::.*  *:*    *. ******:.:*:: ***:***  :****. :

ACEV                    INHVVNKLLIKFDLCNIANQICGTYSGGNKRKLSTCLSLIGYPNCVLLDEPTNGIDPITR 1362
Onthophagus taurus      QEDEIDDLLNKCGLNEYSNKPCGTYSGGNKRKLNTCIALIGHPDIITLDEPTTGVDPQNR 1425
                         :. ::.** * .* : :*: ************.**::***:*: : *****.*:** .*
```

```
ACEV                    NELWDVIEY--IKKKSIVILSSHNVHDCEKLCDNLCILHKGIVKEQ--TSIKKLKNKYCI 1418
Onthophagus taurus      RKIKELINETKLNKKSAIIFTSHSMDECELLCDKLSIMKAGKLETDANLSVPELKEKYNI 1485
                        .:: ::*:   ::*** :*::**.:.:** ***:*.*:: * :: :   *: :**:** *

ACEV                    GHILKLKFYDNKHIKI-EHQLHSNFDTKNIKILSNHDYMITVRILNYNWITIFDIIERIK 1477
Onthophagus taurus      GHIIQMKVKSPQQIDTILEALRESFNNVIIEKIAARNNLLTVSIKNSNWGSIISGMESMK 1545
                        ***:::*. . ::*.   . *:..*:.  *: :: :: ::** * * ** :*:. :* :*

ACEV                    NDTENNIEDYWIMESTLDEALIEIANNKL                                1506
Onthophagus taurus      TH-RNEIVDYMVKESSLEEVFLKVAKG--                                1571
                        .. .*:* ** : **:*:*.::::*:.
```

Figure S7. Comparison of the ABC-transporter protein from virus ACEV and the most similar protein from beetles. Alignment of insectvirus Anomala cuprea entomopoxvirus (ACEV) versus the homologous protein from the beetle *Onthophagus taurus*. The highly conserved Walker A motif is shown in grey. Predicted TM domains are underlined. In the alignment, identical amino acids are indicated by "*", conserved and semi-conserved amino acids are indicated by ":" and ".", respectively.

Figure S8

```
Fr483                        MSETGV-------VTIEQEEKILELGRKNIRGWSLVILSLASLGVVFGDIGTSPLYVLPA     53
Micractinium conductrix      MPGVSVSALWARDADLSAKIEAQEHHRRNASGLRLLALAVSTLGVVYGDIGTSPLYVYGS     60
                             *  ..*       . :. : :  *  *:*  *  *: *::::****:**********  :

Fr483                        IFGELRHQPTENFILGVFSTIFWTITLMVLVKYVWFTLAIDDHGEGGVFALYSIIRRAIT     113
Micractinium conductrix      VFPDGAP-ADANRVLGVASTIFWTITSIVLVKYVVFTMQADDNGEGGIFALYALICRSCN     119
                             :* :       * :*** ******** :****** **:  **:****:****::* *: .

Fr483                        SKPSDFGV-------------DTQEEKIPSKTKDFLENNKWARKVIMGIVITCASLTMAD     160
Micractinium conductrix      IRSGTRMTEADLTLSQYQGGQTRRGSRVAARLRGAQERSATLQTLLLVVVLLASNMIISD     179
                              : .   .               : .:: :: :.  *..   :.::: :*: .:.: ::*

Fr483                        GILTPSISVISATEGIQFHTGISHDTVIFITIGILVGLFSIQFLGTGKVGVIFGPTMLVW     220
Micractinium conductrix      GVLTPAISVVSAIEGIQFQTGISQGAVTGISVGILVALFALQSVGTQRVSFLFAPIMVLW     239
                             *:***:***:** *****:****:.:*  *::****.**::* :** :*..:*.* *::*

Fr483                        FVFNLSVGVYNVTKM--PGVFRAFSPHYMYYFWEEFGSWEAFKLLGEVFLAITGVEALYA     278
Micractinium conductrix      FICNATLGIYNICRFGGPSIFRALSPHYMYYFWSGDA-HAAWRALGNIMLCVTGAEALYA     298
                             *: * ::*:**: ::  *.:***:*********.  .   *:: **:::*.:**.*****

Fr483                        DMGHLNAMSIRISFSAIVYPSLVMNYLGQTAVVLLDYNTSSSLYWSSIPAKLAWPSLAIA     338
Micractinium conductrix      DMGHFNAQSIRVSFLAVVFPSLTLTYLGQTAMILQEPAKAAAAFWESVPTPVLWPVVVLA     358
                             ****:** ***:** *:*:***.:.******::* :  .::: :*.*:*: : ** :.:*

Fr483                        ASAAVIASQALITGTFTIVQQAMHANVFPRVAIFQTNKKHAGQIYIPVVNFALLVGSISV     398
Micractinium conductrix      TGAAIIASQALITGAFSIVQQAMAMNAFPRVTLRHTSKHNIGQVYIPEINWLLMVGALIV     418
                             :.**:*********:*:******  *.****:: :*.*:: **:*** :*: *:**:: *

Fr483                        VLIFQSSSKIVSAYGFAVSIVVVLTHIFFCIVLHIQGR-NKLFSFVFSSFFGVISIAFAA     457
Micractinium conductrix      VVVFKTSASIGNAYGLAVITVMLLDTSLFALVALTAWEWHVAAVGAFWLFFTFVTGTFLS     478
                             *::*::*:.* .***:**  *::*   :*.:*     . :     .*  ** .:: :* :
```

```
Fr483                       SLTIKIPKGAWFSAAIGSALIFVSLVWHRGHRMKVRYIKINRLSARQVFSKPSNNS----     513
Micractinium conductrix     SNMSKIPEGAWFSLALSAVLSCITYLWHWGQRKKLDYVRDNKILLRELYEPDPSQADTAG     538
                            *   ***:***** *:.:.*  :: :** *:* *: *:: *::  *:::.   .::

Fr483                       ----------------------------------------------------KNIVFYNE     521
Micractinium conductrix     GSGAAAAAWHGLLRPSGVSLARSSDSGRGAAEVQPLQRLQLVGSCLPVARVPGIGIYYNE     598
                                                                                   ::***

Fr483                       LTDGIVPAYNQLENLITISGTNNIVLSVRKMTIPRVREDQRFLI--TGYDGVYHVVARYG     579
Micractinium conductrix     LLLGVPPVLERFMSLVPALHEVVIFLTVRVVPVPCVLPQERLLVRQLRFGAMYHVVARYG     658
                            *  *: *. ::: .*:       *.*:** : :* *  ::*:*:    :..:********

Fr483                       YAEIIDHGNCFARKLCQAVNAESSDVVFVMGRTKLLTTNTSFYNKA--VIAMYSLLVKLS     637
Micractinium conductrix     YMDAIDHGEEFVDSILQEIHEYLHPATGI---ADLSA-EAAAANAAHGKLASEDLEKGLD     714
                            * : ****: *. .: * ::     .. :   :.* : :::  * *   :*  .*   *.

Fr483                       SWTTDTFNTPTSKLIIFEASYEI-------------------------------------     660
Micractinium conductrix     HHGDNEGSVHRGAIVRVDAAESLTSATGAQVSSSLALQLLSLRRFSSAPQPRSTGSAGDS     774
                                :  ..  . :: .:*: .:

Fr483                       ------------------------------------------------------------     660
Micractinium conductrix     GMQASGATQRQASGAAGPSLARASTSTLAVPLVGDEGSMHSGKGEAADDGPSPWVQMPSQ     834

Fr483                       ------------------------------------------------------------     660
Micractinium conductrix     VRRVRTHLLGLPEGAPAALDDGLHSLPLPESTQRPPLLPLLRGDSTKSLPLPPAGGSARR     894

Fr483                       ------------------------------------------------------------     660
Micractinium conductrix     TPLYRVQRAMSAASGAQEQQGGGQQAQQAQAQQPQPSPLATPTSTLTPAARARLGQQLWQ     954
```

```
Fr483                       ------------------------------------------------------------      660
Micractinium conductrix     RLQMGRLLHRTSAASAMPTLAEGEQAQPADSDARSSLDGRVAATVRTEDILGSAAPEGEE      1014

Fr483                       ------------------------------------------------------------      660
Micractinium conductrix     ALDTDAARRRLFALQGHAGDAGTPLEDGLAAERRLLLAARQRGVVYLVGKSALCAEPGSS      1074

Fr483                       ------------------------------------------------------------      660
Micractinium conductrix     WLKTFLLESAYAALVANCRPTTALYRVPRQGLLAESCRPAAGALVQLAFSCKGMLTAAEA      1134

Fr483                       ------------------------------------------------------------      660
Micractinium conductrix     AEPMWRMQCQRLGWSLDWLDTLPPGTSTWRYFCARMHARHHLRRLLRSLSRAADPSAWEV      1194

Fr483                       ------------------------------------------------------------      660
Micractinium conductrix     FELYRVVDGQDRERGGIQFFHEARLLSLREAVAAAQERHGPLRLARAFAALAVQRSNGSQ      1254

Fr483                       ------------------------------------------------------------      660
Micractinium conductrix     AAGSGGGDSSSVPPPDDQPSSSSCALPLDDRPKSEDTEQEPAVLLPLSEEMRGRRRFCAD      1314

Fr483                       -----------------------------                                     660
Micractinium conductrix     LRGLIWLASGFHTRCAADSLAVLLQSILT                                     1343
```

Figure S8. Comparison of viral K^+ transporter from virus and its host. Alignment of the Pbi chlorella virus Fr483 potassium transporter versus a homologous protein from the host *Micractinium conductrix* (Chlorella Pbi). The algal protein exhibits a much longer C-terminus (the same is true for other alga potassium transporters). Predicted TM domains are underlined. In the alignment, identical amino acids are indicated by "*", conserved and semi-conserved amino acids are indicated by ":" and ".", respectively.

Figure S9

MQQLYKIRSTHEIENIKGGDYDWLEITKPDDSDLSLVQKRTGLEISTSKLILSSHESSHIEGLTEPDKPLMIVLQYPKMVESNLGDFKE

YATSPIILILSNDGDNNDLITISNHEPSFIAKIQEDSKSLKVPITNKKDIMLLVIYYMSQEYRSILRSLNKDANSLEQSLKTATNNIIF

YHVMSIQKTVSSFLDSLANNQNICEKIENDANYFASEKYTELARSASLALEETSEAARHLDYILDKYTSLVSSIVGNNQLVTINKFTE**W**

GIVLSFLSASFGALGMNYYLPGESSHV**TTILVFATIFASSIWLS**KYIRKLLKGKK

Figure S9. Sequence and topology of Lactobacillus phage magnesium transporter. Shown is the amino acid sequence of the putative magnesium transporter from *Lactobacillus* phage Lfellnf. Predicted transmembrane domains are underlined; however, experimental data indicate 3 TM domains (Smith et al., 1993). The highly conserved GMN motif is shown in yellow.

Figure S10

MQLLDM**VIFAGYIGCIVMAGLVIGW**KKSGTTKSYFMADRS**LPWWILGCAILAANISAEHLIGT**TGSAYAMGI**AIGAFELTGALALVVAA**
WLAIPYFVKNQITTMPQFLAMKYDNR**VRSMFATFWIVVYTLVNLTAVSY**MGALAFTSIGIPLE**VGVWALIGFAVLYSAVGGLSSLV**WTD
FIQ**VGILLTAGIACTWFTLDAYGVHV**GSTSIAGSMVSLYKELPNHFQLVLEPGHPAYEN**IPGILGVVGVFLGSLSYFAF**NQFIVQGALA
AKSVKEAQKGM**VFAAFLKLLMPLVVIIPGIMAY**SMTNGSLTPSDKAYPWLIDNFMP**AGFHGLVMAALFAAIISTLAAIL**NSISTMFTLD
IVKVHKPELSDKTLMKVART**VVVVCGAIGASLAIPFLGNL**DQAY**HFIQEFVGFVTPAMLVIFFAALY**WKTNSN**AAIAVTVFSVAANAIV**
KFTVPELAWLD**RFLMVLVGCGVLYSLFSG**KQVEDAHIAPSK**TLVIGGSIVAIIFFATYITF**A

Figure S10. Sequence and topology of the Aeromonas virus 44RR2 sodium/glusose transporter. Predicted TMs are underlined.

Figure S11

```
Vibrio phage         -MELMTGLVMMASASLFIMYACNSLEQTLDYLGRNMKAGAKGALLMAVASSLPEIMVAFA   59
Vibrio jasicida      MLETIFPFLLIIILACYLLKYSCDTFEQAAGYLGRNFPAGVKGATVNAIGSSMPEMCVVIA   60
                      :* :  ::::  *. :: *:*:::**: .*****: **.*** : *:.**:**: *.:*

Vibrio phage         FL-FSGKPELVLAGVFVTAGSAIFNILLIPAVSILYAGDGQGNKVDSFQLDRKVLSRDTF   118
Vibrio jasicida      CLFWFNDPQLVIVALGVTAGSAIFNGCVIPALSIIVAKDDEGNSVDQIELNKSALLRDVF   120
                      * : ..*:**:..: *********  :***:**: * *.:**.**.::*::..* **.*

Vibrio phage         WLLTVEAIFIYFLGLNVFTIGMAATLIILYALYVSHVIHDSNKAGEQPEGFEFEELDHTE   178
Vibrio jasicida      WVLTAEIALIVCLGFSEFSIWMALLLNVIYLGYAIHLYFDAKKHGGDDDEYEYEEIDDRG   180
                     *:**.*  :*  **:. *:* **  * ::*  *. *: .*::* * : : :*:**:*.

Vibrio phage         TPKFIAWIGYV--LDFNKHLFKNKAYTTKTASIVCALSCTIIAVACHYLAVSTEIVSAAL   236
Vibrio jasicida      ------FIGNLLTFNFNAILFSNKTFTLTRALIVLALAIAVISGSSHILVEGVLGSAAVL   234
                           :** :  ::**  **.**::* . * ** **: ::*: :.* *. ..   :*.*

Vibrio phage         GIPVLIGAAVFAAAATSLPDTILSKSASENGEGDDAVANAVGSNIFDTSFAIGLPLLIAL   296
Vibrio jasicida      GVPEFFSGLVFGAAASSIPDLILSVKDAQKGEYEDAIANPLASNTFDTTVAFALPLFVWF   294
                     *:* ::.. **.***:*:** *** . :::** :**:** :.** ***:.*:.***:: :

Vibrio phage         TPLGEWLFGINLKEGIPLVQGDSFMDTVRYFVIGTSALAAAGLWVQAKNVTKKTAYYLLT   356
Vibrio jasicida      I--------LNGVDSLPMAQD-NNLTILRWSIIGITAAVACSLLFNYKKVTKSVAYFLLA   345
                              :*  :.:*:.*. . :  :*: :** :* .*..* .: *:***..**:**:

Vibrio phage         LYGTWIAYLIYHVS        370
Vibrio jasicida      MFAIWATSMYFVIQ        359
                     ::. * : : : :.
```

Figure S11. Comparison of viral sodium/calcium symporter with most similar protein from a *Vibrio* bacterium. Alignment of vibrio phage 1.084.O._10N.261.49.F5 sodium/calcium symporter versus the most similar protein from *Vibrio jasicida*. Predicted TMs are underlined. In the alignment, identical amino acids are indicated by "*", conserved and semi-conserved amino acids are indicated by ":" and ".", respectively.

Figure S12

```
RSIV            ------------------------------------------------------------
Pagrus major    MSSAEMGKFNISPDEDSSSYSSNSNDFSYPYPTKPAAMKSHYADIDPENQNFLLDSNLGK

RSIV            ------MAPCVLQCGVTDVFLHTLNCMIGIGLLALPHAVAVVGPVVFVGVLLFVAVAAIV
Pagrus major    KKYETQYHPGTTSFGM--SVFNLSNAIVGSGILGLSFAMANTGIALFVILLLFVSIFSLY
                        * . . *:    .::  *.::* *:*.* .*:* .* .:** :****:: ::

RSIV            STHMLTACLRTHRG-NLEDICAAMFGRGGYWIMCAVIILENLGSVCSYMHMLMSVLAVLV
Pagrus major    SVHLLLKTANEGGSLLYEQLGMKAFGMAGKLAASGSITMQNIGAMSSYLFIVKYELPLVI
                *.*:*    .   .   *::    ** .*    .. * ::*:*::.**:.::   * :::

RSIV            P-------DISMYYAVG-----MLILLVLFPVSLPKSSRCLMIISGPALMCVLAFVVYTI
Pagrus major    KTFMNIEETTGEWYLNGDYLVLLVSVILILPLSLLKNLGYLGYTSGFSLLCMVFFLIVVI
                          . :*  *     :: :::::*:** *.   *   ** :*:*:: *:: .*

RSIV            YYCVHA-----------TVPGPAWPLFDDRMRVDQC--------------LSIITFAFVC
Pagrus major    WKMFQIPCPMDSVVMNVTFNATVAPLVDENITIDMCKPKYFIFNSQTVYAVPILTFSFVC
                :  .:            *. . . **.*:.: :* *              : *:**:***

RSIV            QPTIVTTANL-HGDKASGAAAAAMCAMSVGTVLYAVIALCGWLPMGPETPDNIVLAYMAT
Pagrus major    HPAILPIYEELKGRSRKRMMNVSYVSFFAMFLMYLLAALFGYLTFYGKVEPELLHTYSAY
                :*:*:   :  :* . .    .:  :: .  ::* : ** *:* :  :.  ::: :* *

RSIV            LDGKIVAGVM----CVSVVLTVPALLLPVVHMMAPI--DRSPMQWMSTL----GMYACMY
Pagrus major    LGADVLLLIVRLAVLTAVTLTVPVVIFPIRSSITQLLWAGKEFSWLRHCSITVALLAFTN
                *...::  ::     .:*.****.:::*:   :: :    . :.*:       .: *

RSIV            VLVIMIPSFKIGVVVVGAVAGTVLVFALPALMYTWLFYRRRVLAWMV--VVLYTTIGIAV
Pagrus major    VLVIFVPTIRDIFGFIGASAAAMLIFILPSAFYIKLVKKEPMKSVQKIGASFFFLSGILV
```

```
              ****::*:::  . .:** *.::*:* **: :*  *. :. : :      . ::   ** *

RSIV          S------VMAIYDIKIQLQGY
Pagrus major  MTGCMTLIIILDWTQNVTSDGH
                     ::  :  ::  :*:
```

Figure S12. Comparison of viral amino acid transporter with most similar protein from a fish. Alignment of red seabream iridovirus (RSIV) amino acid transporter versus the most similar protein from *Pagrus major* (red seabream). Predicted TMs are underlined. In the alignment, identical amino acids are indicated by "*", conserved and semi-conserved amino acids are indicated by ":" and ".", respectively.
